# Developmental outcome of electroencephalographic findings in *SYNGAP1* encephalopathy

**DOI:** 10.3389/fcell.2024.1321282

**Published:** 2024-03-05

**Authors:** Juliana Ribeiro-Constante, Alba Tristán-Noguero, Fernando Francisco Martínez Calvo, Salvador Ibañez-Mico, José Luis Peña Segura, José Miguel Ramos-Fernández, María del Carmen Moyano Chicano, Rafael Camino León, Víctor Soto Insuga, Elena González Alguacil, Carlos Valera Dávila, Alberto Fernández-Jaén, Laura Plans, Ana Camacho, Nuria Visa-Reñé, María del Pilar Martin-Tamayo Blázquez, Fernando Paredes-Carmona, Itxaso Marti-Carrera, Aránzazu Hernández-Fabián, Meritxell Tomas Davi, Merce Casadesus Sanchez, Laura Cuesta Herraiz, Patricia Fuentes Pita, Teresa Bermejo Gonzalez, Mar O'Callaghan, Federico Felipe Iglesias Santa Polonia, María Rosario Cazorla, María Teresa Ferrando Lucas, Antonio González-Meneses, Júlia Sala-Coromina, Alfons Macaya, Amaia Lasa-Aranzasti, Anna Ma Cueto-González, Francisca Valera Párraga, Jaume Campistol Plana, Mercedes Serrano, Xenia Alonso, Diego Del Castillo-Berges, Marc Schwartz-Palleja, Sofía Illescas, Alia Ramírez Camacho, Oscar Sans Capdevila, Angeles García-Cazorla, Àlex Bayés, Itziar Alonso-Colmenero

**Affiliations:** ^1^ Pediatric Neurology Department Sant Joan de Déu (SJD) Children’s Hospital, Barcelona, Spain; ^2^ Department of Genetics, Microbiology and Statistics, University of Barcelona, Barcelona, Spain; ^3^ Molecular Physiology of the Synapse Laboratory, Institut de Recerca Sant Pau (IR Sant Pau), Universitat Autònoma de Barcelona, Barcelona, Spain; ^4^ Pediatric Neurology Department, Hospital Universitario Miguel Servet, Zaragoza, Spain; ^5^ Pediatric Neurology Department, Arrixaca University Hospital, Murcia, Spain; ^6^ Pediatric Neurology Department - IBIMA Group, Hospital Regional Universitario de Málaga, Málaga, Spain; ^7^ Pediatric Neurology Department, Hospital Universitario Reina Sofía, Córdoba, Spain; ^8^ Pediatric Neurology Department, Hospital Universitario Infantil del Niño Jesús, Madrid, Spain; ^9^ Pediatric Neurology Department, Neurogenetics Section, Hospital Universitario Quironsalud, Madrid, Spain; ^10^ Mental Health in Intellectual Disability Specialized Service Althaia, Xarxa Assistencial, Manresa, Spain; ^11^ Pediatric Neurology Department, Hospital 12 de Octubre, Universidad Complutense de Madrid, Madrid, Spain; ^12^ Paediatric Department, Arnau de Vilanova University Hospital, Lleida, Spain; ^13^ Pediatric Neurology Department, Hospital General Universitario de Jerez de la Frontera, Jerez de la Frontera, Spain; ^14^ Pediatrics Department, Arnau de Vilanova University Hospital, Lleida, Spain; ^15^ Pediatric Neurology Department, Hospital Universitario Donostia, San Sebastian, Spain; ^16^ Pediatric Neurology Department, Complejo Asistencial Universitario de Salamanca, Salamanca, Spain; ^17^ Pediatric Neurology Department, Hospital de Manises, Valencia, Spain; ^18^ Pediatric Neurology Department, Hospital Clínico Universitario Santiago de Compostela, Santiago de Compostela, Spain; ^19^ Pediatric Neurology Department, Sevilla, Spain; ^20^ Neurology Department, Hospital Universitario de Burgos, Burgos, Spain; ^21^ Pediatric Neurology Department, Puerta de Hierro Majadahonda Universitary Hospital, Madrid, Spain; ^22^ Paediatric Department Hospital Universitario Virgen del Rocío, Sevilla, Spain; ^23^ Pediatric Neurology Department, Vall d'Hebron University Hospital, Universitat Autónoma de Barcelona, Bercelona, Spain; ^24^ Department of Clinical and Molecular Genetic Vall d'Hebron University Hospital, Universitat Autónoma de Barcelona, Bercelona, Spain; ^25^ Eurecat, Technology Center of Catalonia, Multimedia Technologies, Barcelona, Spain; ^26^ Center for Brain and Cognition (CBC), Department of Information Technologies and Communications (DTIC), Pompeu Fabra University, Barcelona, Catalonia, Spain; ^27^ Department of Experimental and Health Sciences, Universitat Pompeu Fabra, Barcelona, Spain; ^28^ Pediatric Neurometabolism: Neural Communication Mechanisms and Personalized Therapies Pediatric Neurology Department: Neural Communication Mechanisms and Personalized Therapies Institut de Recerca Sant Joan de Déu, Esplugues de Llobregat, Spain; ^29^ Department of Child Neurology, Epilepsy and Neurophysiology Unit, Member of the ERN EpiCARE, Hospital Sant Joan de Dèu, Barcelona, Spain

**Keywords:** *SYNGAP1*, rare disease, interictal epileptiform discharges, diffuse fast activity, autism spectrum disorder, EEG, disorganized background activity, developmental and epileptic encephalopathy

## Abstract

*SYNGAP1* haploinsufficiency results in a developmental and epileptic encephalopathy (DEE) causing generalized epilepsies accompanied by a spectrum of neurodevelopmental symptoms. Concerning interictal epileptiform discharges (IEDs) in electroencephalograms (EEG), potential biomarkers have been postulated, including changes in background activity, fixation-off sensitivity (FOS) or eye closure sensitivity (ECS). In this study we clinically evaluate a new cohort of 36 SYNGAP1-DEE individuals. Standardized questionnaires were employed to collect clinical, electroencephalographic and genetic data. We investigated electroencephalographic findings, focusing on the cortical distribution of interictal abnormalities and their changes with age. Among the 36 SYNGAP1-DEE cases 18 presented variants in the *SYNGAP1* gene that had never been previously reported. The mean age of diagnosis was 8 years and 8 months, ranging from 2 to 17 years, with 55.9% being male. All subjects had global neurodevelopmental/language delay and behavioral abnormalities; 83.3% had moderate to profound intellectual disability (ID), 91.7% displayed autistic traits, 73% experienced sleep disorders and 86.1% suffered from epileptic seizures, mainly eyelid myoclonia with absences (55.3%). A total of 63 VEEGs were revised, observing a worsening of certain EEG findings with increasing age. A disorganized background was observed in all age ranges, yet this was more common among older cases. The main IEDs were bilateral synchronous and asynchronous posterior discharges, accounting for ≥50% in all age ranges. Generalized alterations with maximum amplitude in the anterior region showed as the second most frequent IED (≥15% in all age ranges) and were also more common with increasing age. Finally, diffuse fast activity was much more prevalent in cases with 6 years or older. To the best of our knowledge, this is the first study to analyze EEG features across different age groups, revealing an increase in interictal abnormalities over infancy and adolescence. Our findings suggest that *SYNGAP1* haploinsufficiency has complex effects in human brain development, some of which might unravel at different developmental stages. Furthermore, they highlight the potential of baseline EEG to identify candidate biomarkers and the importance of natural history studies to develop specialized therapies and clinical trials.

## Introduction

The *SYNGAP1* gene, which is predominantly expressed in forebrain regions (Porter et al., 2005), encodes for a repressor of small GTPases, especially those from the Ras and Rap families (Chen et al., 1998). In adult mammalian brains SynGAP is highly enriched at the postsynaptic density of excitatory synapses (Chen et al., 1998 and Lopez-Rivera et al., 2020), where it is one of the most abundant proteins (Cheng et al., 2006). Because of this, the function of SynGAP is best understood in the context of synaptic biology, where it has a role in AMPA receptor (AMPAR) trafficking, synaptic plasticity and dendritic spine morphology (Komiyama et al., 2002; Kim et al., 2003; Vazquez et al., 2004). *Syngap1* ^+/−^ mice consistently present increased levels of AMPARs at synapses, as well as hyperexcitability and a reduced seizures threshold (Kim et al., 2003; Clement et al., 2012; Kopanitsa et al., 2018). In addition, more recent studies indicate that SynGAP would also have important roles during brain development, contributing to neuronal development (Clement et al., 2012; Gou et al., 2020; Llamosas et al., 2020) or participating in the maturation of neuronal progenitors (Birtele et al., 2023). Thus, SynGAP is an important protein both for neuronal development and adult brain function.

*De novo* loss of function variants in the *SYNGAP1* gene cause an autosomal dominant neurodevelopmental condition (Hamdan et al., 2009), which is currently named *SYNGAP1*-related Developmental and Epileptic Encephalopathy (SYNGAP1-DEE, orphanet code: 544254). Variants in *SYNGAP1* could represent one of the most prevalent monogenic forms of Intellectual Disability (ID) (1 case every 16.000 births, Lopez-Rivera et al., 2020), possibly explaining up to 1% of all ID cases (Hamdan et al., 2009; Mignot et al., 2016). Patients with pathogenic *SYNGAP1* variants display multiple phenotypes with variable penetrance. Yet, developmental delay, moderate or severe ID and generalized epilepsies are present in the vast majority of cases (Mignot et al., 2016; Jimenez-Gomez et al., 2019; Vlaskamp et al., 2019). Other common phenotypes include i) autism spectrum disorder (ASD)/autistic traits (signs or characteristics of autism without meeting all criteria for ASD), occurring in more than half of the affected individuals (Mignot et al., 2016; Vlaskamp et al., 2019), ii) severe sleep disturbances, identified in over 60% of cases (Smith-Hicks et al., 2021; Paasch et al., 2023) or iii) behavioral problems, in more than 70% of cases (Vlaskamp et al., 2019). SYNGAP1-DEE appears to be equally prevalent in males and females and is found in all ethnic groups with similar prevalence.

Almost all subjects described in the literature with SYNGAP1-DEE present one or more forms of generalized seizures. Focal epilepsies are also reported but these are much less common. The age of seizure onset varies widely across the literature and, although it has been found as early as at 3 months of age, it is most commonly reported to start between the ages of 2 and 3 years (Mignot et al., 2016; Agarwal et al., 2019; Vlaskamp et al., 2019). Among the different forms of generalized seizures, SYNGAP1-DEE cases most frequently present eyelid myoclonia with absences, typical and atypical absences and myoclonic-atonic seizures (Mignot et al., 2016; Agarwal et al., 2019; Vlaskamp et al., 2019).

Some studies analyzing EEG recordings describe a certain predominance of IEDs in posterior regions of SYNGAP1-DEE cases, postulating that these might have potential value as biomarkers (Berryer et al., 2013; Mignot et al., 2016; Jimenez-Gomez et al., 2019; Lo Barco et al., 2021). For instance, a slow or absent posterior dominant rhythm (PDR) has been proposed as a potential prognosis marker (Jimenez-Gomez et al., 2019) and fixation-off sensitivity and eye closure sensitivity, which are posterior paroxysms appearing when eyes close, could be primary seizure triggers (Lo Barco et al., 2021). In this study, we have analyzed electroencephalographic findings in 36 individuals with pathogenic or likely pathogenic *SYNGAP1* variants, focusing on the cortical distribution of IEDs and, specially, on the EEG changes observed along developmental time.

## Materials and methods

### Patient recruitment and clinical phenotyping

Individuals were referred through a Spanish network of collaborating child neurologists, geneticists, and psychiatrists, and through Asociación SYNGAP1 España. For one patient, the medical reference could not be reached, so an online interview was conducted with the caregiver. Inclusion criteria encompassed individuals with: i) a diagnosed developmental encephalopathy, either with or without epilepsy, ii) pathogenic or likely pathogenic *SYNGAP1* variants, and iii) with at least one VEEG recording. A standardized phenotypic questionnaire was provided to referring physicians to assess clinical features, genetic pathogenic variants, developmental milestones, and epilepsy features (Supplementary Material).

The severity of intellectual disability (ID) was established with Intelligence Quotient (IQ) scores or information on the level of functioning in accordance with the fifth edition of the Diagnostic and Statistical Manual of Mental Disorders (DSM-5), American Psychiatric Association 2023. Behavioral abnormalities level was established based on the clinical evaluation of neuropediatricians or psychiatrists. Autism spectrum disorder (ASD) was assessed according to DSM-5 autism criteria. Global motor function was scored using the Gross Motor Function (GMF) scale (Palisano et al., 1997). Anthropometric data were converted to standard deviation scores (BMI Z-score and height Z-score) as recommended by the World Health Organization. The Sleep Disturbance Scale for Children (SDSC) (Bruni et al., 1996) was used to identify sleep disorders. Seizure types were classified using the 2017 ILAE classification (Fisher et al., 2017).

### Genetic studies

Genetics studies were performed following standard clinical protocols established in the hospitals participating in this study. Briefly, human *SYNGAP1* variants were identified in DNA isolated from blood samples of affected individuals. In most cases *SYNGAP1* variants were identified through exome sequencing using Illumina sequencing technology. In some instances, gene panels relevant to Neurodevelopmental Disorders (NDDs) were used. All variants identified were individually validated by standard Sanger sequencing with the same DNA samples.

*SYNGAP1* gene variants were annotated and classified with Ensembl Variant Effect Predictor (McLaren et al., 2016), Annovar (Yang and Wang, 2015) and Varsome (Kopanos et al., 2019) using HG38 as the reference human genome. Of note, variants in this cohort that were classified as with unknown significance (VUS) by Varsome were further investigated. In these cases, we analyzed blood DNA samples from the parents using Sanger sequencing to confirm that the variation was *de novo* and reviewed if the variant had been described in the previous literature (Parker et al., 2015; Vlaskamp et al., 2019). Final pathogenicity was stablished following the classification guidelines defined by the American College of Medical Genetics (ACMG) as we added and sum the additional criteria PS2 for *de novo* variants, thus shifting the classification of these variants from VUS to likely pathogenic.

### VEEG collection and analysis

VEEG was performed at the reference medical centers of patients. When possible, VEEG recordings were sent to the Children’s Hospital Sant Joan de Déu (HSJD) for review. VEEGs were obtained with a minimum of 19 electrodes placed according to the 10/20 international system. The average recording time was 30 min, and sleep was observed in 38 of the 63 VEEGs. If the patient had more than one VEEG taken the same year, only the best quality one was included in the study. Thus, only one VEEG per year of life was taken into account for each individual. Initially, VEEGs were individually analyzed by the neurophysiologists or epileptologists of each center according to the standard clinical read (background activity, IEDs, ictal events and other alterations not associated to epileptogenesis). Later, these analyses were reviewed together in a retrospective study performed by the same neurophysiologist and neurologist at HSJD. Furthermore, two pediatric neurologists (HSJD) reviewed personal and family history (including antenatal/perinatal history) and seizures/epilepsy history, development and behavior, as well as physical and neurologic examination. The description of VEEG reports was made according to the glossary of terminology revised in 2017 (Kane et al., 2017) and the current classification of seizures according to the ILAE (Fisher et al., 2017).

### Standard protocol approvals, registrations, and patient consents

All participants’ caregivers gave written informed consent. This study was approved by the local institutional Ethics Committee (Children’s Hospital Sant Joan de Déu ID number: PIC-232-20) and following the Declaration of Helsinki and relevant guidelines and regulations.

### Statistical analysis of the frequency of VEGG abnormalities observed in SYNGAP1-DEE cases

For qualitative analysis of electroencephalographic abnormalities, patients were divided in four age groups, in which EEG findings do not experience large changes in the healthy population (Britton et al., 2016): i) less than 3 years; ii) ≥ 3 and <6 years, iii) ≥ 6 and <10 years and iv) 10 or more years. The most common IEDs and their spatial maximum expression in the cortex were considered for statistical analyses. For the calculations of diffuse fast activity, patients with drugs that have a quantitative effect on EEG were excluded (Hyun et al., 2011). We applied binomial statistics to analyze if the frequency of VEEG abnormalities observed between age groups of SYNGAP1-DEE cases either increased or decreased. The null hypothesis tested was that no change in frequency would occur between age groups. This test was applied to the following VEEG parameters: i) background activity, ii) sleep architecture defined as the presence or absence of spindles, iii) bilateral synchronous and asynchronous occipital discharges, iv) generalized discharges with maximum in the anterior region, v) multifocal and focal IEDs and vi) fast activity.

## Results

### Genetic and clinical phenotyping of the cohort

We introduce a cohort of 36 individuals presenting clinical features compatible with the Developmental and Epileptic Encephalopathy caused by *SYNGAP1*. All cases present heterozygous genetic variants in the *SYNGAP1* locus that are predicted to be pathogenic or likely pathogenic (Table 1 and Supplementary Table S1). The mean age of diagnosis was 8 years and 8 months (range: 2 years −17 years) and 55.9% of this cohort were males. To the best of our knowledge, of the 36 genetic variants present in this cohort, 18 had never been previously reported, nor in the published literature, neither in the curated database of clinical variants ClinVar (Landrum, et al., 2020). Patient 29 presented an additional genomic modification compatible with Klinefelter Syndrome on a 60k array-CGH (arr(X)x2(Y)x1). Yet, this patient was included in the study because his clinical phenotype was consistent with SYNGAP1-DEE.

**TABLE 1:** Genetic Variants Identified in our Cohort of SYNGAP1-DEE Cases and Prediction of Their Pathogenicity.

Patient ID	cDNA variant	Protein variant	Variant type	Exon	Protein domain#	Variant effect predictor	Varsome	*De novo*	Pathogenicity (ACMG)*	Previously reported ^	Publication (author, year)	Existing variant (ClinVar)
**1**	c.403C>T	p.Arg135*	Nonsense	5/19	PH	High	Pathogenic	Not available	Pathogenic	Yes	Mignot et al. (2016)	rs1131692154
**2**	c.2059C>T	p.Arg687*	Nonsense	12/19	GAP	High	Pathogenic	*De novo*	Pathogenic	Yes	Gao et al. (2018)	rs1060503383
**3**	c.3706 C>T	p.Gln1236*	Nonsense	17/19	DAB2P_C	High	Pathogenic	Not available	Pathogenic	Yes		rs1554122729
**4**	c.1393delC	p.Leu465Phefs*9	Frameshift	9/19	GAP	High	Pathogenic	*De novo*	Pathogenic	Yes	Vlaskamp et al. (2019)	rs1057518183
**5**	c.3557C>A	p.Ser1186*	Nonsense	16/19	DAB2P_C	High	Pathogenic	*De novo*	Pathogenic	Yes		rs1554122485
**6**	c.2899C>T	p.Arg967*	Nonsense	15/19	DAB2P_C	High	Pathogenic	*De novo*	Pathogenic	Yes	Vlaskamp et al. (2019)	rs749188610
**7**	c.1726T>C	p.Cys576Arg	Missense	11/19	GAP	Moderate	Likely Pathogenic	*De novo*	Likely pathogenic	No		
**8**	c.1783delC	p.Leu595Lysfs*55	Frameshift	11/19	GAP	High	Pathogenic	*De novo*	Pathogenic	Yes	O'Roak et al. (2014)	rs587780470
**9**	c.3583G>A	p.Val1195Met	Missense - Splice Region	17/19	DAB2P_C	Moderate	VUS	*De novo*	Likely pathogenic*	No		
**10**	c.3526delG	p.Glu1176Lysfs*20	Frameshift	16/19	DAB2P_C	High	Likely Pathogenic	*De novo*	Likely pathogenic	No		
**11**	c.2270delG	p.Gly757Alafs*3	Frameshift	13/19	DAB2P_C	High	Likely Pathogenic	*De novo*	Likely pathogenic	No		
**12**	c.1722delG	p.Arg575Alafs*75	Frameshift	11/19	GAP	High	Likely Pathogenic	*De novo*	Likely pathogenic	No		
**13**	c.857T>C	p.Leu286Pro	Missense	8/19	C2	Moderate	VUS	*De novo*	Likely pathogenic*	No		
**14**	c.1171_1172delGG	p.Gly391Glnfs*27	Frameshift	8/19		High	Likely Pathogenic	*De novo*	Likely pathogenic	No		
**15**	*6p21.32*	Not Applicable	Gene Deletion			High	Not Applicable	*De novo*	Pathogenic	No		
**16**	c.2896delC	p.His966Thrfs*111	Frameshift	15/19	DAB2P_C	High	Likely Pathogenic	*De novo*	Likely pathogenic	No		
**17**	c.1163delG	p. Gly388Alafs*15	Frameshift	8/19		High	Likely Pathogenic	Not available	Likely pathogenic	No		
**18**	c.793A>T	p.Lys265*	Nonsense	8/19	C2	High	Likely Pathogenic	*De novo*	Likely pathogenic	No		
**19**	c.1861C>T	p.Arg621*	Nonsense	11/19	GAP	High	Likely Pathogenic	*De novo*	Likely pathogenic	Yes	Aguilera et al. (2021)	rs1060503386
**20**	c.844T>C	p.Cys282Arg	Missense	8/19	C2	Moderate	VUS	*De novo*	Likely pathogenic*	Yes	Vlaskamp et al. (2019)	rs1581987022
**21**	c.797T>C	p.Leu266Pro	Missense	8/19	C2	Moderate	VUS	*De novo*	Likely pathogenic*	No		
**22**	*6p21.32-p21.31*	Not Applicable	Gene Deletion			High	Not Applicable	*De novo*	Pathogenic	No		
**23**	c.3718C>T	p.Arg1240*	Nonsense	17/19	DAB2P_C	High	Pathogenic	Not available	Pathogenic	Yes	Vlaskamp et al. (2019)	rs869312955
**24**	c.1717C>T	p.Arg573Trp	Missense	11/19	GAP	Moderate	Pathogenic	*De novo*	Pathogenic	Yes	Pekeles et al. (2019)	rs1064795331
**25**	c.881C>A	p.Thr294Asn	Missense	8/19	C2	Moderate	VUS	*De novo*	Likely pathogenic*	No		
**26**	c.1167_1168delAG	p.Gly391Glnfs*27	Frameshift	8/19		High	Pathogenic	Not available	Pathogenic	Yes	Michaelson et al., 2018	rs1060503378
**27**	c.1167_1168delAG	p.Gly391Glnfs*27	Frameshift	8/19		High	Pathogenic	*De novo*	Pathogenic	Yes	Michaelson et al. (2018)	rs1060503378
**28**	c.2091G>A	p.Trp697*	Nonsense	12/19	GAP	High	Likely Pathogenic	*De novo*	Likely pathogenic	No		
**29**	c.2097_2098delGC	p.Leu700Alafs*39	Frameshift	12/19	GAP	High	Likely Pathogenic	Not available	Likely pathogenic	No		
**30**	c.737T>C	p.Leu246Pro	Missense	7/19	PH	Moderate	Likely Pathogenic	*De novo*	Likely pathogenic	No		
**31**	c.509G>A	p.Arg170Gln	Missense	5/19	PH	Moderate	VUS	*De novo*	Likely pathogenic*	Yes	Parker et al. (2015)	rs1057519546
**32**	c.1735C>T	p.Arg579*	Missense	10/19	GAP	High	Likely Pathogenic	Not available	Likely Pathogenic	Yes	Hamdan et al. (2009)	rs121918316
**33**	c.2438delT	p.Leu813Argfs*23	Nonsense	15/19	DAB2P_C	High	Pathogenic	Not available	Pathogenic	Yes	Hamdan et al. (2009)	rs397515320
**34**	c.1861C>T	p.Arg621*	Nonsense	11/19	GAP	High	Likely Pathogenic	*De novo*	Likely Pathogenic	Yes	Aguilera et al. (2021)	rs1060503386
**35**	c.2764C>T	p.Arg922*	Nonsense	15/19	DAB2P_C	High	Pathogenic	*De novo*	Pathogenic	Yes	Parker et al. (2015)	rs1554122244
**36**	c.1221_1224delACAA	p.Thr408*	Nonsense	8/19	PH	High	Likely Pathogenic	*De novo*	Likely pathogenic	No		

# Domains by InterPro. PH: Plekstrin Homology (IPR001849), C2 (IPR000008), GAP: GTPases, activating protein (IPR001936), DAB2P_C: Disabled homolog 2-interacting protein, C-terminal domain (IPR021887).

* Pathogenicity stablished following American College of Medical genetics guidelines (ACMG); In patient 29 an arr(X)x2(Y)x1 (Klinefelter Sd) was identified with a 60k array-CGH.

^ Present in ClinVar or published in previous literature.

**FIGURE 1:**
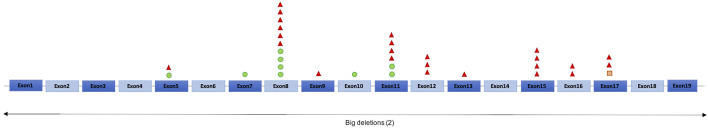
Distribution of pathogenic variants identified in this cohort along the structure of the *SYNGAP1* gene. Pathogenic variants reported in this study indicated above the corresponding exon in which they are found. Green circle: missense variants; orange square: splicing variant; red triangle: truncating variants (stop codon and frameshift).

Twenty-six of these variants, representing almost 75% of the total, are loss of function variants, that is they either: i) create a stop codon (Nonsense variants in Table 1, 13 cases), ii) delete of 1 or 2 nucleotides causing a frameshift that results in a downstream stop codon (Frameshift variants in Table 1, 11 cases) or iii) are big deletions encompassing the entire *SYNGAP1* locus (Gene Deletion in Table 1, 2tbl2 cases). Of the two cases with large deletions, one affects 5 genes (*KIFC1, PHF1, CUTA, SYNGAP1 and ZBTB9*) and the other deletion affects 10 genes including SYNGAP1. The remaining 10 variants are missense, changing the aminoacidic sequence. Of note, we found two variants repeated in two independent cases. Cases 19 and 34 both had the nonsense variant c.1861C>T, and cases 26 and 27 shared a deletion of two nucleotides (c.1167_1168delAG). We identified disease related variants in 12 of the 19 exons of the *SYNGAP1* gene. Exons at 5′ and 3′ ends presented no pathogenic variants (Figure 1). We found 21 pathogenic variants in exons 8 to 12, which encode for the PH, C2 and GAP domains (Figure 1; Table 1). Exon 8 was specially populated with pathogenic variants having 10. Thus, more than half of the variants identified concentrated in 5 of the 19 exons of the gene, which account for 1/3 of the base pairs of the canonical *SYNGAP1* isoform (ENST00000455687.6).

Several *in silico* prediction tools were used to investigate the pathogenicity of these variants, including Ensembl Variant Effect Predictor (McLaren et al., 2016), Annovar (Yang and Wang, 2015) and Varsome (Kopanos et al., 2019). Nevertheless, the final pathogenicity status was established following ACMG guidelines (see Table 1). Based on these, we identified 15 Pathogenic and 21 Likely Pathogenic variants (Table 1). We established that all variants in this cohort classified as with unknown significance (VUS) by Varsome were *de novo*, because of this, and following ACMG guidelines we could finally classify these as Likely pathogenic (6 variants marked with* in Table 1).

Most members of this cohort were born at term (34 out of 36 patients) and all had normal birthweight for gestational age. All patients had global neurodevelopmental and language delays, as well as behavioral abnormalities. Intellectual disability (ID) was reported in all cases, albeit with different degrees: 11.7% had mild ID, 32.3% had moderate ID, 41.2% had severe ID and 14.7% had profound ID. Autistic traits were present in 91.7% of the cases in this cohort and 73% of the subjects experienced sleep disorders (Supplementary Table S1). Regarding behavioral abnormalities, aggression was observed in 72.7%, anxiety in 75.7%, flat affect in 37.5%, impulsiveness in 72.7% and inattentiveness in 87.9% of all cases.

**FIGURE 2:**
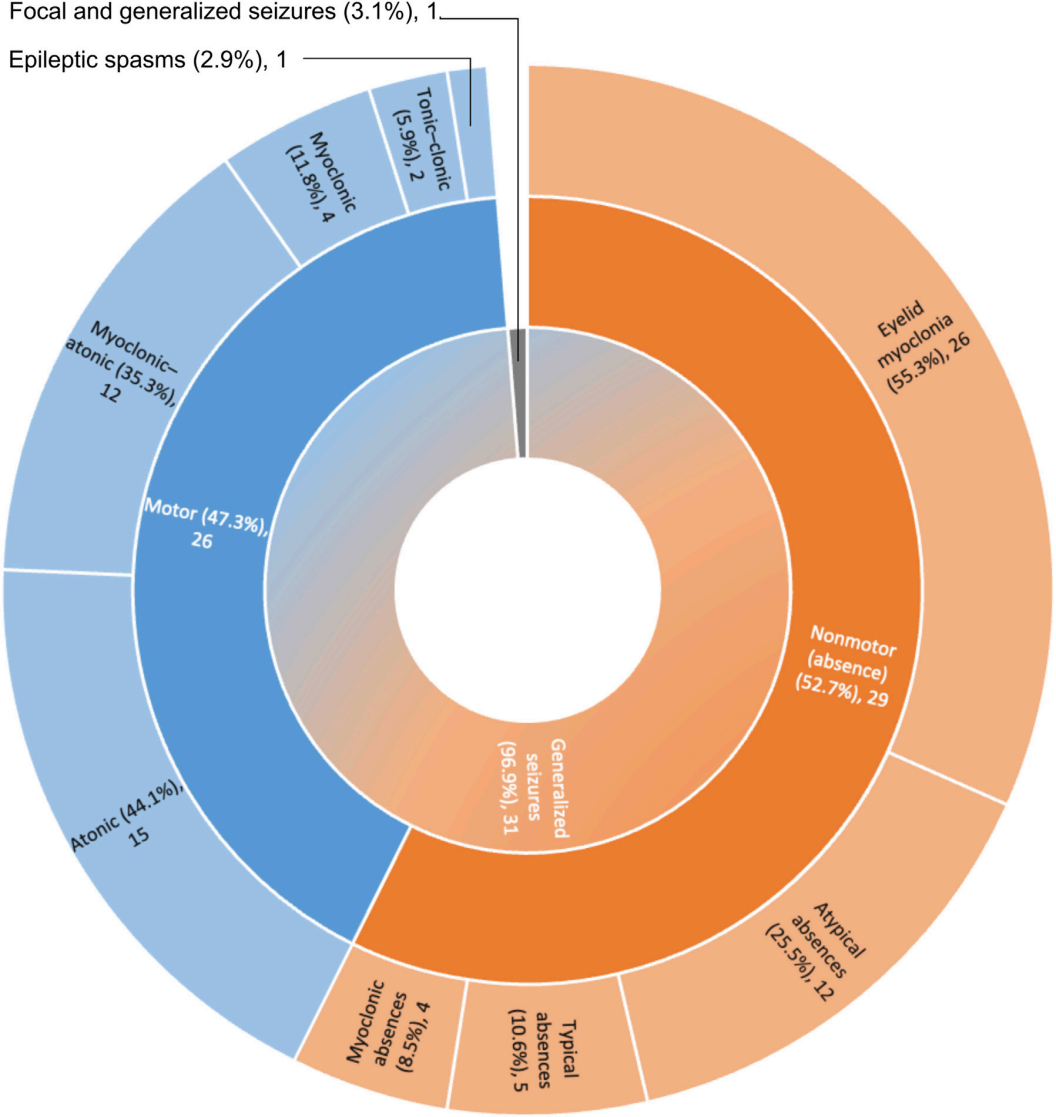
Classification of seizure types in the SYNGAP1-DEE cohort. N = 29; Motor seizures: 2; Nonmotor seizures: 3; Motor and Nonmotor seizures: 27.

Thirty-one patients had generalized seizures (96.9%), featuring eyelid myoclonia (55.3%), myoclonic-atonic seizures (35.3%), atonic seizures (44.1%) and atypical absences (25.5%) (Figure 2). In our cohort the proportion of subjects with pharmacoresistant epilepsy was found to be smaller when compared with previously published cohorts, as this was close to 40% (Supplementary Table S1), while previous reports indicate frequencies of up to 60% (Mignot et al., 2016; Vlaskamp et al., 2019).

### VEEG findings observed along infancy, adolescence and young adulthood in SYNGAP1-DEE

Sixty-three VEEGs were analyzed, with 2 in the <3 years group, 20 in the 3 to less than 6 years group, 19 in the 6 to less than 10 years group, and 22 in the 10 or more years group. At least one scalp VEEG was performed in each case (range: 1 - 6 per case). The mean age of VEEG recordings was 8.2 years (range 2y-18y). Archived VEEG clips of seizures were available for 15 individuals and standardized epilepsy questionnaires, prepared according to the ILAE current classification of seizures, were performed in the remaining 21 cases. We collected multiple EEGs for each year of life between the ages of 2–16, while for ages 17, 18 and 20 we only had one recording. Seizures were registered in 12 VEEGs, all had generalized non-motor seizures, 9 had eyelid myoclonias with/without absence, 2 had absence seizures (typical or atypical) and 1 had both types of seizures. Fixation-off sensitivity (FOS) was observed in one patient, eye closure sensitivity (ECS) in 5 and photoparoxysmal response in 6. Two subjects had a combination of both, ECS with FOS or photoparoxysmal response (Supplementary Table S2).

**FIGURE 3:**
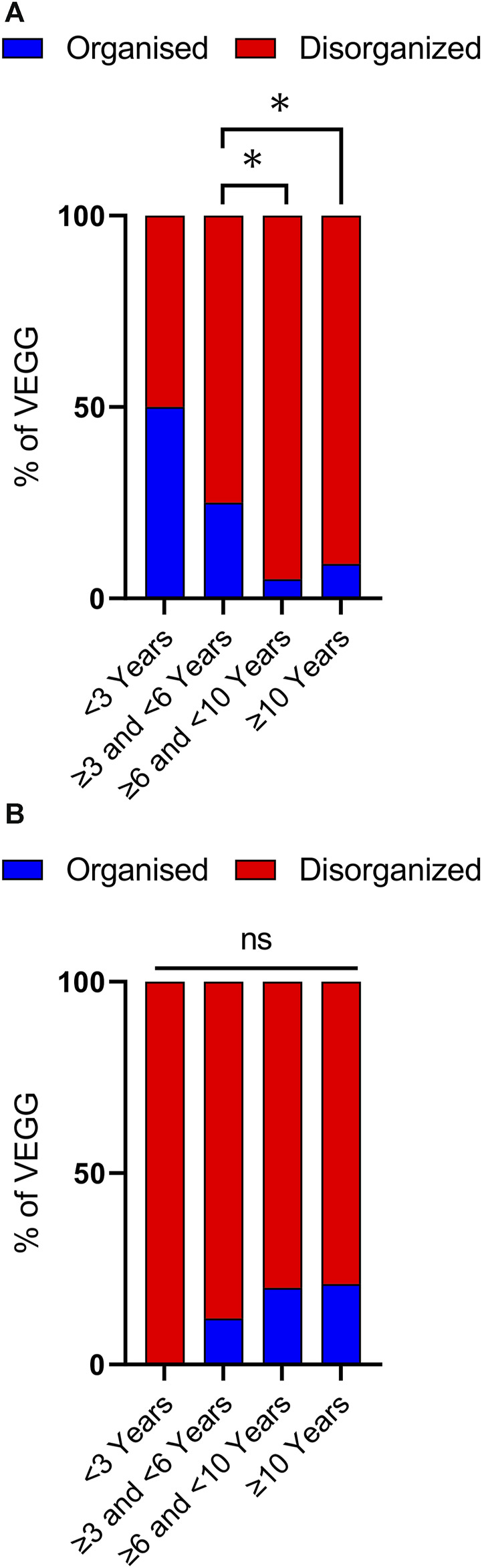
Analysis of background activity and sleep architecture along developmental time. **(A)** Background Activity in patients with *SYNGAP1* pathogenic variants. Total VEEGs reviewed: 63. 28 reviews of specific reports and questionnaires; 35 reviews of VEEG clips. Cases per age group: i) < 3 years, 2; ii) ≥ 3 and <6 years, 20; iii) ≥ 6 and <10 years, 18 and iv) 10 or more years, 22. **(B)** Sleep architecture according to age. Total VEEGs reviewed: 38. Cases per age group: i) < 3 years, 1; ii) ≥ 3 and <6 years, 7; iii) ≥ 6 and <10 years, 15 and iv) 10 or more years, 14. Differences in frequencies between age groups were analyzed using binomial statistics, *p < 0.05.

**FIGURE 4:**
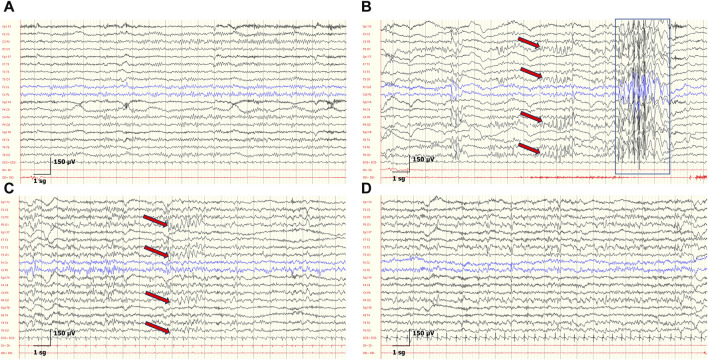
Developmental EEGs changes and IEDs in the patient P6. **(A)** At 3 years, background activity is organized according to age. **(B)** At 4 years, background activity is organized according to age. Generalized spike-and-wave discharges with anterior maximum (blue rectangle) and bilateral spike-and-wave discharges with posterior maximum, indicated by red arrows in the corresponding electrode pairs. **(C)** At 7 years, disorganized background according to age, and bilateral spike-and-wave complex with posterior maximum (red arrows). **(D)** At 8 years, disorganized background according to age, and abnormal diffuse rapid activity. Sensitivity: 150 μV, LFF: 0.3 Hz, HFF: 50 Hz.

Disorganized background activity (DBA), understood as a gross alteration in the frequency, shape, topography, and/or amount of physiological EEG rhythms compared to findings in normal subjects of similar age and state of alertness (Kane et al., 2017), was observed in 51 out of 63 VEEG recordings, covering all age ranges. Nevertheless, DBA was more frequently observed in older age groups (Figures 3, 4). In the group of cases with 3 to less than 6 years of age DBA was observed in 75% of cases, while in the group with 6 to less than 10 years this increased to 95% and to 91% in the last age group (Figure 3A). Indeed, only 3 out of 41 EEGs recorded in children with 6 or more years, could be classified as having an organized background activity. Binomial statistics shows that the frequency of DBA is significantly higher in both older age groups as compared with cases between 3 and less than 6 years of age.

Sleep was recorded in 38 of 63 VEEGs (Supplementary Table S2), allowing us to assess the age-dependent organization of sleep architecture by analyzing the presence or absence of sleep spindles (Kahn et al., 1996; Schönauer and Pöhlchen, 2018; Fernandez and Lüthi, 2020). These graphoelements are the hallmark of the N2 stages of NREM sleep and evolve with a characteristic profile throughout life that parallels cortical maturation in early postnatal periods, throughout adolescence and aging (Kane et al., 2017). We observed a very clear predominance of disorganized sleep architecture in all age groups (Figure 3B). Absence of sleep spindles was found in 89% of cases younger than 6 years, in 80% of patients between 6 and 10 years, and in 79% of cases in the 10 years and older group. No statistically significant differences were observed between the groups (binomial statistics).

**FIGURE 5:**
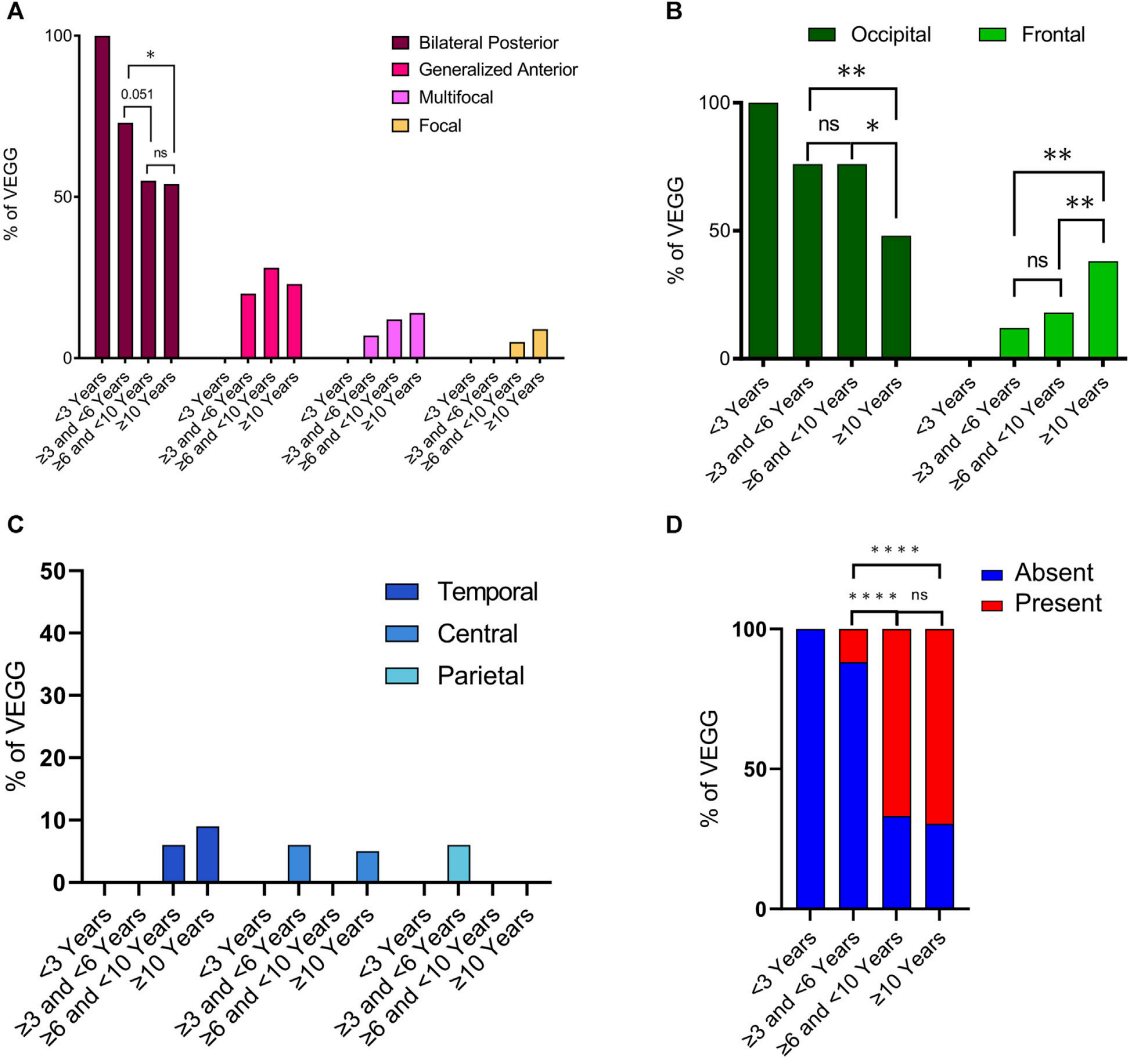
Characterization of interictal epileptiform discharges along developmental time. Total VEEGs reviewed: sixty-three (63). Twenty-eight (28) reviews of specific reports and questionnaires; 35 reviews of video-EEG clips. **(** **A)** Frequency of main interictal epileptiform abnormalities in different age groups; N = 57. Bilateral Posterior refer to “bilateral synchronous and asynchronous occipital discharges” and Generalized Anterior refer to “generalized discharges with maximum in the anterior region”. **(B)** Frequency of epileptiform alterations in VEEGs in different age groups for occipital and frontal cortices. N = 57. **(C)** Frequency of epileptiform alterations in VEEGs in different age groups for temporal, central and parietal cortices. N = 57. **(D)** Frequency of VEEGs with Diffuse Fast Activity in different age groups N = 58. Differences in frequencies between age groups were analyzed using binomial statistics, ****p < 0,0001; **p < 0.001; *p < 0.05, ns not significant.

Interictal epileptiform discharges (IEDs) were observed in 57 out of 62 VEEGS (information was not available in one EEG recording). Of the 5 VEEGs where IEDs were absent, 4 were in infants less than 6 years old (Supplementary Table S2). IEDs were classified into 4 types: i) “bilateral synchronous and asynchronous occipital discharges”, which was by far the most frequent IED (found in more than 50% of cases in all age ranges); ii) “generalized discharges with maximum amplitude in the anterior region”, the second interictal alteration in frequency, iii) multifocal and iv) focal IEDs (Figure 5A). When comparing the frequency of these IED types between age groups we observed that bilateral occipital discharges were less prevalent in cases 6 years or older. While discharges with a maximum in the anterior region had an opposed trend, appeared and worsened with age. Statistical analysis indicates that the reduction in bilateral posterior discharges between the group of 3 to ≤6 years and that of 6 to ≤10 is very close to significance (*p* = 0.051, binomial statistics) and the comparison between cases of 3 to ≤6 years and the group with the oldest cases is statistically significant (*p* = 0.028, binomial statistics). On the other hand, there is no difference in the frequency of these IEDs between the two groups of older cases. Indicating that the reduction in bilateral occipital discharges occurs between the ages of 6 and 9 and remains stable thereof. Concomitant to this reduction in occipital features, we observed an increase of generalized IEDs in anterior regions with age. Although this trend did not reach statistical significance.

To further investigate the changes in spatial distribution of IEDs with age we quantified their maximum expression, that is their maximum amplitude, in 5 cortical regions (Frontal, Temporal, Central, Parietal and Occipital). Overall, IEDs mostly appeared in frontal or occipital regions (Figure 5B), as only 1 EEG presented alterations in the parietal region, 2 in the central region and 3 in the temporal cortex (Figure 5C). In line with the analysis of IED types, we observed a clear reduction of aberrant activity in the occipital region along time (Figure 5B, dark green columns), although it did not completely disappear. In parallel, we observed the appearance and a clear increase in frontal discharges with age (Figure 5B, light green columns). These differences were more noticeable when comparing the group of 3 to ≤6 years with that of 10 years or older (*p* = 0.0015 for frontal cortex, *p* = 0.0029 for occipital cortex; binomial statistics). Indeed, there was no statistical difference when comparing cases in the second and third age groups for neither of the two cortical regions (Figure 5B). Instead, statistical significance was found when comparing the group of 6 to less than 10 years with the group of older cases (*p* = 0.015 in frontal cortex, *p* = 0.0029 in occipital cortex; binomial statistics). Finally, we also looked into diffuse fast activity, and we observed that this was much more frequent with increasing age. A clear increase in the frequency of EEGs presenting diffuse fast activity was detected when comparing the cases in the age group 3 to ≤6 years with those in the group of 6 to ≤10 years (*p* = 8,4e-07), or the group of 10 or older (*p* = 1,4e-10). The presence of this EEG feature was actually very rare in children younger than 6, as it was only identified in 2 out of 19 recordings. No statistical difference was observed between the two older age groups (Figure 5D).

We also investigated if there was a statistically significant correlation between the severity of clinical symptoms (including behavior abnormalities, ID, gross motor abilities and refractory epilepsy) and EEG findings (including background activity, maximum expression of IEDs or spatial distribution of the epileptiform discharges). No correlation could be detected for any of those comparisons between any of the age groups.

## Discussion

In this study we introduce a new cohort of 36 SYNGAP1-DEE patients recruited through a network of Spanish neuropaediatricians. We have performed an extensive clinical phenotyping of this series, with especial emphasis on EEG features. We analyzed electroencephalographic characteristics across different age groups, ranging from 2 to 20 years, showing an increase in interictal abnormalities from infancy to young adulthood.

We used different bioinformatics methods to re-evaluate the pathogenicity of all *SYNGAP1* variants in the cohort, which were initially characterized by more than 20 different Hospitals, confirming that all were pathogenic or likely pathogenic. Of note, 18 of these variants had, to the best of our knowledge, never been previously reported in the literature. We identified 27 loss of function variants, which represented close to 75% of the total. Of these, two were large deletions encompassing the entire *SYNGAP1* locus and the rest resulted in premature stop codons. On the other hand, missense variants account for the remaining 25% of the variants. Similar proportions of these variant types are reported in Varsome for a much larger number of variants. The effect of missense variants on protein function or stability is difficult to predict. On the one hand they could result in a reduction of functional protein, yet it is also plausible that *SYNGAP1* alleles with missense variants are expressed at normal levels. If the latter is correct, other pathological mechanisms, beyond that of haploinsufficiency (Hamdan et al., 2009), might co-exist in SYNGAP1-DEE.

Overall, the main clinical phenotypes identified in this cohort, and their prevalence are well in line with previous reports (Mignot et al., 2016; Agarwal et al., 2019; Jimenez-Gomez, et al., 2019; Vlaskamp et al., 2019). All individuals displayed intellectual disability language delay and behavioral abnormalities. Additionally, sleep disorders, autism or autistic traits, and epilepsy were observed in most individuals. The main clinical difference between this cohort and previously reported ones would be the prevalence of cases diagnosed with autism spectrum disorders, which reached 73.5%, while the literature describes this to be observed in approximately half of the affected individuals. As a matter of fact, in total, 92% of our cohort was reported to present autistic traits. The relevance of this discrepancy would deserve further investigation, although it might be due to the extensive neuropsychological characterization of our series. If this is the case, the prevalence of autism among individuals with SYNGAP1-DEE might have been underestimated in previous studies.

Regarding epilepsy, 31 cases presented generalized seizures, featuring eyelid myoclonia, myoclonic-atonic seizures, atonic seizures and atypical absences. Furthermore, a number of cases presented with photosensitivity, FOS and ECS. All these findings are also in good agreement with the previous literature (Mignot et al., 2016; Agarwal et al., 2019; Jimenez-Gomez, et al., 2019; Vlaskamp et al., 2019) and indicate that the main symptoms of SYNGAP1-DEE are already well defined, as they are reproducible between cohorts from different countries and genetic ancestries. In our cohort the frequency of refractory epilepsies was found to be close to 40%, which is less than what has been reported in other studies (Mignot et al., 2016; Vlaskamp et al., 2019).

Having a cohort with an extended age range and having performed EEGs in all its members allowed us to investigate the temporal development of EEG features in SYNGAP1-DEE, with the goal to better understand the course of this condition. As EEG features change along normal development, we divided EEGs into age groups where these remain quite constant (Britton et al., 2016). We defined 4 age groups of patients: i) those less than 3 years of age, ii) cases ≥3 and <6 years, iii cases) ≥ 6 and <10, and finally, iv) a group of cases with 10 or more years.

We first investigated the organization of EEG background activity. Developmental changes in background activity serve as a clear expression of functional brain maturation and connectivity (Gmehlin et al., 2011). Furthermore, background activity is an important parameter to evaluate brain dysfunction (Staba &Worrell, 2014), although its physiological mechanism is still poorly understood. During infancy, there is a progressive development of occipital alpha rhythms (8-12 Hz) while awakened and the appearance of vertex sharp transients and sleep spindles during sleep (Oka et al., 2021). Conversely, poor sleep architecture has been observed in refractory epilepsy and epileptic encephalopathies (Proost et al., 2022), and there is a progressive increase in slow wave activities in encephalopathies, whether the cause is metabolic, septic, toxic or structural, the extent of which parallels the severity of the brain dysfunction (Smith, 2005).

Similar to what has been reported in the literature, in our cohort we observed that background activity, which could even be identified in cases of only 2 years of life, was disorganized in the majority of EEGs from SYNGAP1-DEE cases. Importantly, we observed that disorganized background activity (DBA) was more common in older age groups. This difference, which was statistically significant, was particularly remarkable if patients of 6 years or older were considered together, as only 3 out of 41 EEGs presented an organized background activity. This worsening of DBA with age might suggest that SynGAP protein function during nervous system development becomes increasingly complex or fundamental. Previous studies have shown a link between neurophysiological abnormalities and developmental outcomes. Among genetic neurodevelopmental encephalopathies, diffuse background slowing appears to increase with age in Dravet syndrome, an infantile-onset developmental and epileptic encephalopathy due to pathogenic variants in the *SCN1A* gene (Minato and Myers, 2021). In Rett syndrome, a progressive neurological disorder due to pathogenic variants in the *MECP2* gene, a progressive slowing of background activity is a hallmark of disease evolution (Glaze, 2005). Furthermore, DBA is observed in other encephalopathies, such as in inborn errors of metabolism, both in situations of acute encephalopathy and in neurodegenerative diseases (van Karnebeek et al., 2018). For instance, in an international study of argininosuccinate lyase deficiency, present with progressive intoxication due to accumulation of ammonia in the body, generalized background slowing was found in twelve out of nineteen patients (63%) (Elkhateeb et al., 2023). In turn, in neuronal ceroid lipofuscinoses (NCLs), a heterogeneous group of autosomal recessive neurodegenerative disorders, the EEG is marked by a progressive decline in cerebral activity, characterized by deceleration of background activity and the vanishing of spindles during sleep (Trivisano et al., 2022).

As sleep disorders are present in more than 60% of the *SYNGAP1* population (Vlaskamp et al., 2019), we also examined sleep architecture by analyzing sleep spindles, an EEG signature that reflects the strength and malleability of thalamocortical circuits (Schönauer and Pöhlchen, 2018; Fernandez and Lüthi, 2020). As expected, most EEGs reflected a disorganized sleep architecture with reduced spindle activity in 82% of the VEEGs investigated. Reduced sleep spindles have been correlated with cognitive dysfunction and psychotic events (Schönauer and Pöhlchen, 2018; Fernandez and Lüthi, 2020). Their reduction may be of clinical relevance both for behavior and cognitive function in SYNGAP1-DEE.

We next investigated interictal epileptiform discharges (IED). The presence of epileptiform discharges with an occipital distribution is very common in infants and young children. They are found in 0.9% of normal preschool children and in 0.1% of typical older children (Datta et al., 2021). These alterations occur in epilepsies of variable etiology and prognosis, ranging from structural, genetic, infectious, metabolic, autoimmune or even of unknown etiology. Less commonly, they occur in other forms of epilepsy, such as genetic generalized epilepsy (Datta et al., 2021). The spatio-temporal expression of IED is consistent with brain maturation and tends to disappear or move to middle temporal regions in older children (Adcock and Panayiotopoulos, 2012). Studies analyzing EEG recordings on SYNGAP1-DEE cases describe a certain predominance of occipital epileptiform discharges (Berryer et al., 2013; Mignot et al., 2016; Jimenez-Gomez 2019; Lo Barco et al., 2021). The rationale for this phenomenon is not clear, since occipital epileptiform discharges in *SYNGAP1* patients are mainly observed at 2-3 years, after the onset of epilepsy (Mignot et al., 2016; Jimenez-Gomez 2019; Lo Barco et al., 2021), by which time the critical period of neuronal myelination has already ended (Gilmore et al., 2018). A possible explanation for this phenomenon would be the hyperexcitability of the visual cortex observed in *Syngap1* ^+/−^ mice (Katsanevaki, 2018; Kilinc et al., 2018). The fact that FOS, ECS and photosensibility are quite common in SYNGAP1-DEE strengthens this hypothesis, as these phenomena are an expression of occipital hyperexcitability (Koutroumanidis et al., 2009; Vaudano et al., 2017).

We also observed that the frequency of IEDs in anterior regions increase with age, especially in the frontal cortex. In our group of cases of 10 years or older the frequency of occipital and frontal epileptiform alterations as the main IED was 48% and 38% respectively, while in the age group of ≥3 and <6 years, occipital alterations accounted for 77%, while frontal only represented 18% of all IEDs. Furthermore, it is interesting to note that of the 62 VEEG recordings, 5 did not present any IEDs, of which 4 were recorded before the age of 6 years. These observations indicate that there is a deterioration in the electroencephalographic pattern of SYNGAP1-DEE cases.

To the best of our knowledge, this study is the first to show the presence of diffuse fast rhythms in SYNGAP1-DEE. We found that diffuse fast activity is almost completely absent before the age of 6, while afterwards it becomes highly predominant. The relationship between different forms of hyperexcitability and the development of abnormal neuronal rhythms is not yet fully understood, and the generation of excessive rhythmic activity in different frequency bands, with or without epileptic discharges, may support the diagnosis of genetic syndromes (Alfei et al., 2014). For example, high-amplitude rhythmic slow-wave activity has long been recognized as a feature of Angelman Syndrome; increased fast rhythms, beta and gamma waves, have been extensively reported in Fragile X Syndrome (Goodspeed et al., 2023), and high amplitude fast activity is often seen in patients with Beta-propeller Protein-Associated Neurodegeneration (BPAN), (Kidokoro et al., 2020), and Lissencephaly Syndrome (Alfei, et al., 2014). These observations suggest that increased rhythmic alpha-beta activity may be an EEG pattern reflecting undetectable neuroradiological malformations or dysfunctional circuitry (Alfei et al., 2014).

This study has some limitations, mainly the relatively small number of cases in the cohort. A longitudinal study might have given more insight in the developmental progression of EEG findings in SYNGAP1-DEE. Furthermore, for several patients we only had access to VEEG reports, and a large part of neuropsychological assessments were based on the clinical impressions of referring physicians.

In conclusion, this study is the first to describe VEEGs changes occurring over the years in SYNGAP1-DEE. We observed a disorganized background activity that seems to worsen with age and a possible change in the pattern of IEDs, which shows an increasing involvement of anterior regions, especially the frontal cortex. Finally, we also observed that diffuse fast activity is much more frequent in older cases. These findings could suggest that *SYNGAP1* haploinsufficiency has increasingly complex functions in human brain development that unfold along development. Or, alternatively, they could be the consequence of a highly disruptive distinctive event occurring early in neurodevelopment, that has a long-lasting negative impact on brain development. Clarifying which of these two hypotheses is correct is something that will require further investigation. Our findings support the potential of baseline EEG to identify candidate biomarkers and reinforces the importance of natural history studies to develop specialized therapies and clinical trials for SYNGAP1-DEE.

## Data Availability

The original contributions presented in the study are included in the article/Supplementary Material, further inquiries can be directed to the corresponding authors.

## Abbreviations

ASD, Autism spectrum disorder; BW, Birthweight; ECS, Eye closure sensitivity; EEG, electroencephalogram; DBA, disorganized background activity; DEE, developmental and epileptic encephalopathy; FOS, Fixation-off sensitivity; GMF, Gross Motor Function; ID, Intellectual Disability; IEDs, Interictal Epileptiform Discharges; IQ, Intelligence Quotient; VEEG, Video electroencephalograms; PDR, Posterior dominant rhythm.
