# Correction: Developmental outcome of electroencephalographic findings in SYNGAP1 encephalopathy

**DOI:** 10.3389/fcell.2026.1824815

**Published:** 2026-05-18

**Authors:** Juliana Ribeiro-Constante, Alba Tristán-Noguero, Fernando Francisco Martínez Calvo, Salvador Ibañez-Mico, José Luis Peña Segura, José Miguel Ramos-Fernández, María del Carmen Moyano Chicano, Rafael Camino León, Víctor Soto Insuga, Elena González Alguacil, Carlos Valera Dávila, Alberto Fernández-Jaén, Laura Plans, Ana Camacho, Nuria Visa-Reñé, María del Pilar Martin-Tamayo Blázquez, Fernando Paredes-Carmona, Itxaso Marti-Carrera, Aránzazu Hernández-Fabián, Meritxell Tomas Davi, Merce Casadesus Sanchez, Laura Cuesta Herraiz, Patricia Fuentes Pita, Teresa Bermejo Gonzalez, Mar O’Callaghan, Federico Felipe Iglesias Santa Polonia, María Rosario Cazorla, María Teresa Ferrando Lucas, Antonio González-Meneses, Júlia Sala-Coromina, Alfons Macaya, Amaia Lasa-Aranzasti, Anna Ma Cueto-González, Francisca Valera Párraga, Jaume Campistol Plana, Mercedes Serrano, Xenia Alonso, Diego Del Castillo-Berges, Marc Schwartz-Palleja, Sofía Illescas, Alia Ramírez Camacho, Oscar Sans Capdevila, Angeles García-Cazorla, Àlex Bayés, Itziar Alonso-Colmenero

**Affiliations:** 1 Pediatric Neurology Department Sant Joan de Déu (SJD) Children’s Hospital, Barcelona, Spain; 2 Department of Genetics, Microbiology and Statistics, University of Barcelona, Barcelona, Spain; 3 Molecular Physiology of the Synapse Laboratory, Institut de Recerca Sant Pau (IR Sant Pau), Universitat Autònoma de Barcelona, Barcelona, Spain; 4 Pediatric Neurology Department, Hospital Universitario Miguel Servet, Zaragoza, Spain; 5 Pediatric Neurology Department, Arrixaca University Hospital, Murcia, Spain; 6 Pediatric Neurology Department- IBIMA Group, Hospital Regional Universitario de Málaga, Málaga, Spain; 7 Pediatric Neurology Department, Hospital Universitario Reina Sofía, Córdoba, Spain; 8 Pediatric Neurology Department, Hospital Universitario Infantil del Niño Jesús, Madrid, Spain; 9 Pediatric Neurology Department, Neurogenetics Section, Hospital Universitario Quironsalud, Madrid, Spain; 10 Mental Health in Intellectual Disability Specialized Service Althaia, Xarxa Assistencial, Manresa, Spain; 11 Pediatric Neurology Department, Hospital 12 de Octubre, Universidad Complutensede Madrid, Madrid, Spain; 12 Paediatric Department, Arnau de Vilanova University Hospital, Lleida, Spain; 13 Pediatric Neurology Department, Hospital General Universitario de Jerez de la Frontera, Jerez dela Frontera, Spain; 14 Pediatrics Department, Arnau de Vilanova University Hospital, Lleida, Spain; 15 Pediatric Neurology Department, Hospital Universitario Donostia, San Sebastian, Spain; 16 Pediatric Neurology Department, Complejo Asistencial Universitario de Salamanca, Salamanca, Spain; 17 Pediatric Neurology Department, Hospital de Manises, Valencia, Spain; 18 Pediatric Neurology Department, Hospital Clínico Universitario Santiago de Compostela, Santiago de Compostela, Spain; 19 Pediatric Neurology Department, Sevilla, Spain; 20 Neurology Department, Hospital Universitario de Burgos, Burgos, Spain; 21 Pediatric Neurology Department, Puerta de Hierro Majadahonda Universitary Hospital, Madrid, Spain; 22 Paediatric Department Hospital Universitario Virgen del Rocío, Sevilla, Spain; 23 Pediatric Neurology Department, Vall d’Hebron University Hospital, Universitat Autónoma de Barcelona, Bercelona, Spain; 24 Department of Clinical and Molecular Genetic Vall d’Hebron University Hospital, Universitat Autónoma de Barcelona, Bercelona, Spain; 25 Eurecat, Technology Center of Catalonia, Multimedia Technologies, Barcelona, Spain; 26 Center for Brain and Cognition (CBC), Department of Information Technologies and Communications (DTIC), Pompeu Fabra University, Barcelona, Spain; 27 Department of Experimental and Health Sciences, Universitat Pompeu Fabra, Barcelona, Spain; 28 Pediatric Neurometabolism: Neural Communication Mechanisms and Personalized Therapies Pediatric Neurology Department: Neural Communication Mechanisms and Personalized Therapies Institut de Recerca Sant Joan de Déu, Esplugues de Llobregat, Spain; 29 Department of Child Neurology, Epilepsy and Neurophysiology Unit, Member of the ERN EpiCARE, Hospital Sant Joan de Dèu, Barcelona, Spain

**Keywords:** *SYNGAP1*, rare disease, interictal epileptiform discharges, diffuse fast activity, autism spectrum disorder, EEG, disorganized background activity, developmental and epileptic encephalopathy

There was a mistake in Table 1 as published. Typographic errors were found in the following fields: Row 8, column 3 – Row 9, all columns – Row 10, column 2 – Row 14, columns 2, 8, 10 – Row 16, column 2 – Row 32, column 4 – Row 33, column 4 – Row 36, column 2 and 6. The corrected Table 1 appears below.

**TABLE 1:** Genetic Variants Identified in our Cohort of SYNGAP1-DEE Cases and Prediction of Their Pathogenicity.

Patient ID	cDNA variant	Protein variant	Variant type	Exon	Protein domain#	Variant effect predictor	Varsome	*De novo*	Pathogenicity (ACMG)*	Previously reported ^	Publication (Author, Year)	Existing variant (ClinVar)
1	c.403C>T	p.Arg135*	Nonsense	5/19	PH	High	Pathogenic	Not available	Pathogenic	Yes	Mignot (2016)	rs1131692154
2	c.2059C>T	p.Arg687*	Nonsense	12/19	GAP	High	Pathogenic	*De novo*	Pathogenic	Yes	Gao (2018)	rs1060503383
3	c.3706 C>T	p.Gln1236*	Nonsense	17/19	DAB2P_C	High	Pathogenic	Not available	Pathogenic	Yes	​	rs1554122729
4	c.1393delC	p.Leu465Phefs*9	Frameshift	9/19	GAP	High	Pathogenic	*De novo*	Pathogenic	Yes	Vlaskamp (2019)	rs1057518183
5	c.3557C>A	p.Ser1186*	Nonsense	16/19	DAB2P_C	High	Pathogenic	*De novo*	Pathogenic	Yes	​	rs1554122485
6	c.2899C>T	p.Arg967*	Nonsense	15/19	DAB2P_C	High	Pathogenic	*De novo*	Pathogenic	Yes	Vlaskamp (2019)	rs749188610
7	c.1726T>C	p.Cys576Arg	Missense	11/19	GAP	Moderate	Likely pathogenic	*De novo*	Likely pathogenic	No	​	​
8	c.1783delC	p.Leu595Cysfs*55	Frameshift	11/19	GAP	High	Pathogenic	*De novo*	Pathogenic	Yes	O'Roak (2014)	rs587780470
9	c.3583-6G>A	p.Val1195Alafs*27	Frameshift	​	​	Low	Likely pathogenic	Not available	Likely pathogenic	Yes	Lo Barco (2021)	rs869312674
10	c.3524delG	p.Glu1176Lysfs*20	Frameshift	16/19	DAB2P_C	High	Likely pathogenic	*De novo*	Likely pathogenic	No	​	​
11	c.2270delG	p.Gly757Alafs*3	Frameshift	13/19	DAB2P_C	High	Likely pathogenic	*De novo*	Likely pathogenic	No	​	​
12	c.1722delG	p.Arg575Alafs*75	Frameshift	11/19	GAP	High	Likely pathogenic	*De novo*	Likely pathogenic	No	​	​
13	c.857T>C	p.Leu286Pro	Missense	8/19	C2	Moderate	VUS	*De novo*	Likely pathogenic*	No	​	​
14	c.1167_1168delAG	p.Gly391Glnfs*27	Frameshift	8/19	​	High	Pathogenic	*De novo*	Pathogenic	Yes	Michaelson (2018)	​
15	*6p21.32*	Not Applicable	Gene deletion	​	​	High	Not Applicable	*De novo*	Pathogenic	No	​	​
16	c.2895delC	p.His966Thrfs*111	Frameshift	15/19	DAB2P_C	High	Likely pathogenic	*De novo*	Likely pathogenic	No	​	​
17	c.1163delG	p. Gly388Alafs*15	Frameshift	8/19	​	High	Likely pathogenic	Not available	Likely pathogenic	No	​	​
18	c.793A>T	p.Lys265*	Nonsense	8/19	C2	High	Likely pathogenic	*De novo*	Likely pathogenic	No	​	​
19	c.1861C>T	p.Arg621*	Nonsense	11/19	GAP	High	Likely pathogenic	*De novo*	Likely pathogenic	Yes	Aguilera (2021)	rs1060503386
20	c.844T>C	p.Cys282Arg	Missense	8/19	C2	Moderate	VUS	*De novo*	Likely pathogenic*	Yes	Vlaskamp (2019)	rs1581987022
21	c.797T>C	p.Leu266Pro	Missense	8/19	C2	Moderate	VUS	*De novo*	Likely pathogenic*	No	​	​
22	*6p21.32-p21.31*	Not Applicable	Gene deletion	​	​	High	Not Applicable	*De novo*	Pathogenic	No	​	​
23	c.3718C>T	p.Arg1240*	Nonsense	17/19	DAB2P_C	High	Pathogenic	Not available	Pathogenic	Yes	Vlaskamp (2019)	rs869312955
24	c.1717C>T	p.Arg573Trp	Missense	11/19	GAP	Moderate	Pathogenic	*De novo*	Pathogenic	Yes	Pekeles (2019)	rs1064795331
25	c.881C>A	p.Thr294Asn	Missense	8/19	C2	Moderate	VUS	*De novo*	Likely pathogenic*	No	​	​
26	c.1167_1168delAG	p.Gly391Glnfs*27	Frameshift	8/19	​	High	Pathogenic	Not available	Pathogenic	Yes	Michaelson (2018)	rs1060503378
27	c.1167_1168delAG	p.Gly391Glnfs*27	Frameshift	8/19	​	High	Pathogenic	*De novo*	Pathogenic	Yes	Michaelson (2018)	rs1060503378
28	c.2091G>A	p.Trp697*	Nonsense	12/19	GAP	High	Likely pathogenic	*De novo*	Likely pathogenic	No	​	​
29	c.2097_2098delGC	p.Leu700Alafs*39	Frameshift	12/19	GAP	High	Likely pathogenic	Not available	Likely pathogenic	No	​	​
30	c.737T>C	p.Leu246Pro	Missense	7/19	PH	Moderate	Likely pathogenic	*De novo*	Likely pathogenic	No	​	​
31	c.509G>A	p.Arg170Gln	Missense	5/19	PH	Moderate	VUS	*De novo*	Likely pathogenic*	Yes	Parker (2015)	rs1057519546
32	c.1735C>T	p.Arg579*	Nonsense	10/19	GAP	High	Likely pathogenic	Not available	Likely pathogenic	Yes	Hamdan (2009)	rs121918316
33	c.2438delT	p.Leu813Argfs*23	Frameshift	15/19	DAB2P_C	High	Pathogenic	Not available	Pathogenic	Yes	Hamdan (2009)	rs397515320
34	c.1861C>T	p.Arg621*	Nonsense	11/19	GAP	High	Likely pathogenic	*De novo*	Likely pathogenic	Yes	Aguilera (2021)	rs1060503386
35	c.2764C>T	p.Arg922*	Nonsense	15/19	DAB2P_C	High	Pathogenic	*De novo*	Pathogenic	Yes	Parker (2015)	rs1554122244
36	c.1221_1224delGACA	p.Thr408*	Nonsense	8/19	GAP	High	Likely pathogenic	*De novo*	Likely pathogenic	No	​	​

^#^ Domains by InterPro. PH: Plekstrin Homology (IPR001849), C2 (IPR000008), GAP: GTPases activating protein (IPR001936), DAB2P_C: Disabled homolog 2-interacting protein, C-terminal domain (IPR021887).

^*^ Pathogenicity stablished following American College of Medical genetics guidelines (ACMG); In patient 29 an arr(X)x2,(Y)x1 (Klinefelter Sd) was identified with a 60k array-CGH.

^^^ Present in ClinVar or published in previous literature.

File Supplementary Table 1 was erroneously published with the original version of this paper. The file has now been replaced. This new version updates the same typographic errors reported in Table 1.

In the abstract, in the first line of the second paragraph a wrong value was reported: “Among the 36 SYNGAP1-DEE cases 18 presented variants…. ”. This has been corrected to read: “Among the 36 SYNGAP1-DEE cases 16 presented variants…. ”

A correction has been made to the section **Results**, sub-section *Genetic and clinical phenotyping of the cohort*, second paragraph:

“Twenty-eight of these variants, representing **over** 75% of the total, are loss of function variants, that is they either: i) create a stop codon (Nonsense variants in Table 1, 13 cases), ii) delete of 1 or 2 nucleotides causing a frameshift that results in a downstream stop codon (Frameshift variants in Table 1, **13** cases) or iii) are big deletions encompassing the entire SYNGAP1 locus (Gene Deletion in Table 1, 2 cases). Of the two cases with large deletions, one affects 5 genes (*KIFC1*, *PHF1*, *CUTA*, *SYNGAP1* and *ZBTB9*) and the other deletion affects 10 genes including *SYNGAP1*. The remaining **8** variants are missense, changing the aminoacidic sequence. Of note, we found two variants repeated in **multiple** independent cases. Cases 19 and 34 both had the nonsense variant c.1861C>T, and cases **14,** 26 and 27 shared a deletion of two nucleotides (c.1167_1168delAG). We identified disease related variants in **11** of the 19 exons of the *SYNGAP1* gene. Exons at 5′ and 3′ ends presented no pathogenic variants (Figure 1). We found 21 pathogenic variants in exons 8 to 12, which encode for the PH, C2 and GAP domains (Figure 1; Table 1). Exon 8 was specially populated with pathogenic variants, having 10. Thus, more than half of the variants identified concentrated in 5 of the 19 exons of the gene, which account for 1/3 of the base pairs of the canonical *SYNGAP1* isoform (ENST00000455687.6).”

A correction has been made to the section **Results**, sub-section *Genetic and clinical phenotyping of the cohort*, third paragraph:

“Several *in silico* prediction tools were used to investigate the pathogenicity of these variants, including Ensembl Variant Effect Predictor (McLaren et al., 2016), Annovar (Yang and Wang, 2015) and Varsome (Kopanos et al., 2019). Nevertheless, the final pathogenicity status was established following the guidelines of the ACMG (see Table 1). Based on these, we identified 16 Pathogenic and 21 Likely Pathogenic variants (Table 1). We established that all variants in this cohort classified as with unknown significance (VUS) by Varsome were *de novo*, because of this, and following ACMG guidelines we could finally classify these as Likely pathogenic (5 variants marked with* in Table 1).”

A correction has been made to the section **Discussion**, second paragraph:

“We used different bioinformatics methods to re-evaluate the pathogenicity of all *SYNGAP1* variants in the cohort, which were initially characterized by more than 20 different Hospitals, confirming that all were pathogenic or likely pathogenic. Of note, 16 of these variants had, to the best of our knowledge, never been previously reported in the literature. We identified 28 loss of function genetic variants, two causing large deletions encompassing the entire *SYNGAP1* locus or variants that result in premature stop codons, these represent over 75% of the total. On the other hand, missense variants account for the remaining 25% of the variants. Similar proportions of these variant types are reported in Varsome for a much larger number of variants. While the effect on protein function or stability of missense variants is difficult to predict, and these could result in a reduction of functional SynGAP protein, it is also plausible that *SYNGAP1* alleles with missense variants will be expressed at normal levels. This would mean that other pathological mechanisms, beyond that of haploinsufficiency originally proposed (Hamdan et al., 2009), might co-exist in SYNGAP1-DEE.“

The original article has been updated.

