# Supplementary material for: Developmental outcome of electroencephalographic findings in SYNGAP1 encephalopathy
Source: Front Cell Dev Biol. 2024 Mar 5;12:1321282. doi: 10.3389/fcell.2024.1321282 (PMC10948473; doi:10.3389/fcell.2024.1321282)
Supplement: Supplementary file 2 [file DataSheet1.pdf]

Natural History of SYNGAP1 encephalopathy

Diagnosis

Patient identification: ……………………………………..  Date: …… /……. /…….. (DD/MM/YYYY)

Actual age: ………………………………………

# DIAGNOSIS

Diagnosis (choose one): Age at diagnosis of SYNGAP1 mutation (i.e when diagnosis was confirmed by a lab study)

- Prenatal (first month of life); age: _____days
- Newborn (first month of life); age: _____days
- Infant (2nd to 18th month of life); age: _____months
- Child (>18 month to 11 years); age: _____years
- Adolescent/adult (>11 years); age: _____years

Birth day: ______________________________________________________________

Age: ______________________________________________________________

Gender: ______________________________________________________________

RESULTS OF VARIANT ANALYSIS

| SPECIMEN | Nucleotide change (NM_007327) (e.g. c.2539C>T) | Protein change (e.g. p.Arg847Ser, or R847S) | Zygosit (het, hom, hem, not known)" |
|---|---|---|---|
| Proband | | | |
| Mother | | | |
| Father | | | |

Inheritance

- De Novo mutation
- Mother
- Father
- Parents not tested

Functional classification

- Loss of fuction (LOF)
- Gain of function (GOF)

- Complex

Members of the family affected: ___________________________________________________

Clinical follow-up

Country, Hospital: _______________________________________________________________

Previous publication of patient

- Yes. Reference ________________________________________________________
- No
- Unknown

Natural History of SYNGAP1 encephalopathy

# Medical History (Intake)

Patient (ID): ..........................................

Date: ...... /....... /........ (DD/MM/YYYY)

Actual age: ..............................................

Maternal age: _________ years

Paternal age: _________ years

Ethnic origin:

- White
- Asian
- Arabic
- Ashkenazi Jewish
- Black
- Gypsy
- Other

Parental consanguinity:

- Yes
- No
- Unknown

Maternal medical problems during pregnancy (Choose one or more)

- Yes
- No
- Unknown

- Diabetes mellitus
- Arterial hypertension
- Hepatic dysfunction
- Cardiac dysfunction
- Renal dysfunction
- Epilepsy
- Psychiatric disease
- HELLP syndrome
- Thyroid disorder

- Others (add free text):

Therapy during pregnancy (Mother)

- NO
- Yes
- Unknown

If yes, please specify

- Antiepileptics
- Neuroleptics
- Antidepressants
- Thyroid supplements
- Others (add free text):

Delivery (Choose one):

- Spontaneous vaginal delivery
- Induced vaginal delivery
- Vacuum extraction
- Caesarean section

Gestational age at birth: ________________weeks

Anthropometrical parameters at birth.

1. Weight: __________kg Percentile ______________________
2. Length: __________cm Percentile ______________________
3. Head circumference: ________ cm Percentile ______________________

Postnatal problems (under age of 28 days)

- NO
- YES
- Unknown

If yes, please specify

- Hypoxic ischemic encephalopathy
- Mechanical ventilation (including CPAP)
- Hyperbilirubinemia without kernicterus

- Hyperbilirubinemia with kernicterus
- Feeding problems
- Neonatal sepsis
- Hypoglycemia
- Others, please specify

# DEVELOPMENTAL MILESTONES

| Motor development | Achieved (age in months) | Achieved age unknown | Not yet achieved | Not done |
|---|---|---|---|---|
| Head control | | | | |
| Sitting without support | | | | |
| Walking alone without assistance | | | | |
| Climbs on furniture and begins to run | | | | |
| Manipulate objects | | | | |
| Builds a tower of nine or more blocks | | | | |

| Language | Achieved (age in months) | Achieved age unknown | Not yet achieved | Not done |
|---|---|---|---|---|
| Babbles repetitive syllables, such as ""ba, ba, ba | | | | |
| Says a few words, such as ""mama"" and ""uh-oh"" | | | | |
| Says simple phrases (two or three words together) | | | | |
| Points to an object or picture when it's named | | | | |
| Speaks 50 words and understands more | | | | |

| | | | | |
|---|---|---|---|---|
| Alternative Communication | | | | |

| Adaptative Function | Achieved (age in months) | Achieved age unknown | Not yet achieved | Not done |
|---|---|---|---|---|
| Self-feeding | | | | |
| Self-Grooming | | | | |
| Self-Dressing | | | | |
| Writing | | | | |

**DISEASE RELATED SYMPTOM**

1. Patient start of first symptoms (y) ________________________________________

2. Psychomotor retardation
   - ○ Yes    If yes, age of onset (in y) _________________________
   - ○ No

3. Intellectual disability
   - ○ No
   - ○ Yes    If yes
     - o Mild
     - o Moderate
     - o Severe
     - o Profound

4. Psychomotor Regression/Loss of acquired skills
   - ○ Yes    If yes, age of onset (in y) _________________________
   - ○ No    Specify____________________________________

5. Language delay
   - o Yes    If yes, age of onset (in y) _________________________
   - o No

6. If yes to 5, expressive language is affected as much as receptive language
   - o Yes    If yes, age of onset (in y) _________________________
   - o No

7. If yes to 5, expressive language is more affected that receptive language
   - o Yes    If yes, age of onset (in y) _________________________
   - o No

8. Current language ability
   - o Absence of speech
   - o Speaks words
   - o Associates words or simple sentences
   - o Complex sentences

9. Microcephaly
   - ○ Yes    If yes, age of onset (in y) _________________________
   - ○ No

10. Purposeful hand skills
- Yes
- No If no, age of loss (in y) ___________________________

## EPILEPSY

11. Epileptic Seizures
- Yes If yes, age of onset (in y) _________________________
- Only EEG abnormalities
- No

If actually is seizure-free, specify period: ____________________________________________

12. If yes, initial seizure type (Choose one)
- Multiple seizure types
- Generalized seizures
  - Nonmotor seizures
    - Atypical absences
    - Eyelid myoclonia
    - Typical absences
    - Myoclonic absences
  - Motor
    - Myoclonic
    - Myoclonic-atonic
    - Myoclonic–tonic–clonic
    - Tonic-clonic
    - Atonic
    - Tonic
- Focal seizures
  - Motor onset
  - Nonmotor onset
- Other seizure types: spasms
- Unknown seizure types

Please, describe the initial seizure type:
_____________________________________________________________________________
_____________________________________________________________________________
_____________________________________________________________________________
_____________________________________________________________________________

13. If yes, type of seizures during the evolution (Choose one or more)
- Multiple seizure types
- Generalized seizures
  - Nonmotor seizures

        - Atypical absences
        - Eyelid myoclonia
        - Typical absences
        - Myoclonic absences
    - Motor
        - Myoclonic
        - Myoclonic-atonic
        - Myoclonic–tonic–clonic
        - Tonic-clonic
        - Atonic
        - Tonic
- Focal seizures
    - Motor onset
    - Nonmotor onset
- Other seizure types: spasms
- Unknown seizure types

Please, describe the type of seizures during the evolution:
________________________________________________________________________________
________________________________________________________________________________
________________________________________________________________________________
________________________________________________________________________________

14. Frequency
- Rare event (<once a month)
- Frequent (> once a month)
- ____ x per day/ week/ month
- Only with fever
- Unknown

15. Length of seizures:
- 1 minute or less
- 1-5 minutes
- 5-30 minutes
- Status epilepticus
- Unknown
-

16. Status epilepticus
- Yes  If yes, age of onset (in y) __________________________
- No

17. Developmental plateauing or regression occurred with seizures

- Yes If yes, age of onset (in y) _________________________
- No

18. If yes 17, choose one
- Epileptic Encephalopathy with Continuous Spike and Wave During Sleep (CSWS)
- Hypsarrhythmia
- Burst-suppression pattern
- (Multi)focal
- Focal and generalized
- Generalized

19. EEG (Choose one or more)
- Slow background
- EEG: generalized discharges
- EEG: (multi)focal discharges

20. Refractory seizures
- Yes If yes, age of onset (in y) _________________________
- No

21. Photosensitivity seizures (clinical)
- Yes
- No

22. Photosensitivity (only electric changes)
- Yes
- No

23. Reflex seizures
- Yes If yes specify (such as eating, sound, touch…)_____________________
- No ___________________________________________________________

**MOTOR**

24. Muscular Symptoms
- Yes If yes, age of onset (in y) _________________________

- No

Arms: If yes, age of onset (in y) ________________________

- Hypotonia
- Hypertonia

Legs: If yes, age of onset (in y) ________________________

- Hypotonia
- Hypertonia

Trunk: If yes, age of onset (in y) ________________________

- Hypotonia
- Hypertonia

25. Gross motor Function
- Independent
- Orthotics
- Walkers
- Wheelchair

26. Spasticity
- Yes If yes, age of onset (in y) _________________________
- No

27. Abnormal movement
- Yes If yes, age of onset (in y) ________________________
- No

28. Dystonia
- Yes If yes, age of onset (in y) _________________________
- No If yes, specify area______________________________

  - Occasional (<25% of the time)
  - Intermittent (25–50% of the time)
  - Frequent (50–75% of the time)
  - Constant (>75% of the time)

29. Dyskinesia
- Yes If yes, age of onset (in y) _________________________
- No

30. Choreiform movement

- o Yes — If yes, age of onset (in y) ___________________________
- o No

31. Ataxia

- o Yes — If yes, age of onset (in y) ___________________________
- o No

32. Hyperkinetic movements

- o Yes — If yes, age of onset (in y) ___________________________
- o No

33. Myoclonus

- o Yes — If yes, age of onset (in y) ___________________________
- o No

34. Tremor

- o Yes — If yes, age of onset (in y) ___________________________
- o No

**BEHAVIOUR**

35. Sleep Disorders

- o Yes — If yes, age of onset (in y) ___________________________

- No

36. Poor visual contact
- Yes — If yes, age of onset (in y) __________________________
- No

37. Inattention
- Yes — If yes, age of onset (in y) __________________________
- No

38. Hyperactive
- Yes — If yes, age of onset (in y) __________________________
- No

39. Anxiety
- Yes — If yes, age of onset (in y) __________________________
- No

40. Friendliness
- Yes — If yes, age of onset (in y) __________________________
- No

41. Autism Spectrum Disorder
- Yes — If yes, age of onset (in y) __________________________
- No

42. Deficits in social communication
- Yes — If yes, age of onset (in y) __________________________
- No

43. Restricted, repetitive patterns of behavior
- Yes — If yes, age of onset (in y) __________________________
- No

44. Stereotyped or repetitive motor movements
- Yes — If yes, age of onset (in y) __________________________
- No

If yes,

- Hand-washing
- Hand-wringing
- Hand-mouthing stereotypical movements
- Bruxism
- Inappropriate laughter and crying

- Hyperventilation
- Other: ____________________________________________________________

45. Insistence on sameness
- Yes — If yes, age of onset (in y) _________________________
- No

46. Highly restricted, fixated interests
- Yes — If yes, age of onset (in y) _________________________
- No

47. Hyper- or hyporeactivity to sensory input
- Yes — If yes, age of onset (in y) _________________________
- No — If yes, specify witch sensory input _________________

48. High pain threshold.
- Yes — If yes, age of onset (in y) _________________________
- No — If yes, specify one/more examples________________

49. Obsessive-Compulsive
- Yes — If yes, age of onset (in y) _________________________
- No

50. Aggression
- Yes — If yes, age of onset (in y) _________________________
- No

51. Self-injurious bahavior
- Yes — If yes, age of onset (in y) _________________________
- No

52. Oppositional behavior
- Yes — If yes, age of onset (in y) _________________________
- No

53. Tantrums
- Yes — If yes, age of onset (in y) _________________________
- No

54. Hallucinations
- Yes — If yes, age of onset (in y) _________________________
- No

**ORGANS**

**55.** Strabismus

- Yes If yes, age of onset (in y) ___________________________
- No

56. Oculogyric crisis

- Yes If yes, age of onset (in y) ___________________________
- No

57. Cortical visual impairment

- Yes If yes, age of onset (in y) ___________________________
- No

58. Hearing impairment

- Yes If yes, age of onset (in y) ___________________________
- No

59. Oromotor dysfunction. Feeding difficulties

- Yes If yes, age of onset (in y) ___________________________
- No

60. If yes Disphagya

- PO (thickeners)
- Nasogastric tube
- G-tube

61. Drooling

- Yes If yes, age of onset (in y) ___________________________
- No

62. Constipation

- Yes If yes, age of onset (in y) ___________________________
- No

63. Espinctres control

- Yes If yes, age of onset (in y) ___________________________
- No

64. Growth Restriction

- Yes If yes, age of onset (in y) ___________________________
- No

65. Scoliosis
   - o Yes                                        If yes, age of onset (in y) ___________________________
   - o No

**EDUCATION LEVEL/ PROFESSIONAL CAREER**

| | Kindergarten | School |
|---|---|---|
| No | | |
| Yes regular school | | |
| Yes, special educational needs | | |
| Not applicable | | |

| | Professional training | Employment |
|---|---|---|
| No | | |
| Yes | | |
| Not applicable | | |

66. IQ testing ___________________________________________________________________
    a. If yes, age performed (in y) ____________________________________________
    b. Name of the test ______________________________________________________

67. Adaptive behavior testing ______________________________________________________
    a. If yes, age performed (in y) _____________________________________
    b. Name of the test ______________________________________________________

| Natural History of SYNGAP1 encephalopathy | Physical and Neurological Exam |
|---|---|

Patient (ID): ………………………………….. Date: …… /……. /…….. (DD/MM/YYYY)

**Physical and Neurological Exam**

| Anthropometrics and Vital Signs | |
|---|---|
| Height: | cm |
| Weight: | kg |
| Body mass index: | |
| Head circumference: | cm DS |
| Body temperature: | °C |
| Blood pressure: | Systolic: mmHg |
| | Diastolic: mmHg |
| Pulse: | bpm |
| Respiratory rate: | rpm |

| Physical Exam | |
|---|---|
| General state of health | o Not examined<br>o Normal<br>o Abnormal<br>*Please specify* ____________________ |
| Skin appearance | o Not examined<br>o Normal<br>o Abnormal<br>*Please specify* ____________________ |
| Dysmorphic features | o Not examined<br>o Normal<br>o Abnormal<br>*Please specify* ____________________ |

| | |
|---|---|
| Heart sounds | ○ Not examined<br>○ Normal<br>○ Abnormal<br>*Please specify* ________________________________________________ |
| Respiration (auscultation) | ○ Not examined<br>○ Normal |
| | ○ Abnormal<br>*Please specify* ________________________________________________ |
| Ear, nose and throat | ○ Not examined<br>○ Normal ○ Abnormal<br>☐ *Nasal stuffiness*<br>☐ *Other abnormalities. Please specify*________________________________ |
| Abdomen | ○ Not examined<br>○ Normal<br>○ Abnormal<br>*Please specify* ________________________________________________ |
| Feeding tube | ○ Absent<br>○ Present |

| **Sexual Development** | |
|---|---|
| Tanner stage | ○ Not examined<br>○ Examined<br>Stage: _______________________________ |
| Female (if applicable) | Breasts _______(stage 1-5)<br>Pubic hair _______ (stage 1-5)<br>Menarche ○ No ○ Yes, at the age of ________ years |
| Male (if applicable) | Penis ______ (stage 1-5)<br>Pubic hair ______ (stage 1-5)<br>Testes descended ○ No ○ Yes |

| **Neurological Exam** | |
|---|---|
| Language | Expressive<br>◯ Nonverbal<br>◯ Minimal utterances<br>◯ Limited output<br>◯ Mild impairment<br>◯ Normal<br><br>Receptive<br>◯ Mild impairment<br>◯ Moderate impairment<br>◯ Marked impairment<br><br>Alternative Communication<br>◯ Yes<br>◯ No |
| Head control | ◯ Not examined<br>◯ Normal<br>◯ Abnormal<br>*Please specify* ___________________________________ |
| Muscle tone | ◯ Not examined<br>◯ Normal<br>◯ Abnormal<br><br>☐ Arms<br>◯ Hypotonia ◯ Hypertonia<br>Dystonia ☐<br>Spasticity ☐<br>☐ Legs<br>◯ Hypotonia ◯ Hypertonia<br>Dystonia ☐<br>Spasticity ☐<br>☐ Trunk |

| | |
|---|---|
| | ◯ Hypotonia ◯ Hypertonia<br>Dystonia ☐<br>Spasticity ☐ |
| Dystonia | ○ No<br>○ Yes<br><br>If yes, specify area________________________________<br><br>○ Occasional (<25% of the time)<br>○ Intermittent (25–50% of the time)<br>○ Frequent (50–75% of the time)<br>○ Constant (>75% of the time) |
| Movements | ◯ Not examined<br><br>◯ Normal<br><br>◯ Abnormal, specify types and severities of the movement disorders (MD) |

| | Location | Mild | Moderate | Severe |
|---|---|---|---|---|
| Ataxia | Arms | ◯ | ◯ | ◯ |
| | Legs | | | |
| | Trunk | | | |
| ☐ Action tremor | Arms | ◯ | ◯ | ◯ |
| | Legs | | | |
| ☐ Rest tremor | Arms | ◯ | ◯ | ◯ |
| | Legs | | | |
| ☐ Dyskinesia | Arms | ◯ | ◯ | ◯ |
| | Legs | | | |
| | Other | | | |
| ☐ Chorea | Arms | ◯ | ◯ | ◯ |
| | Legs | | | |
| | Other: | | | |
| ☐ Myoclonus | Arms | ◯ | ◯ | ◯ |
| | Legs | | | |

| | |
|---|---|
| | <table><tr><td></td><td>Other</td><td></td><td></td><td></td></tr><tr><td>☐ Hyperkinetic movements</td><td></td><td>○</td><td>○</td><td>○</td></tr><tr><td>☐ Hypokinesia</td><td></td><td>○</td><td>○</td><td>○</td></tr></table> |
| Spine deformity | ○ Absent<br>○ Present<br><br>☐ Lordosis<br>☐ Kyphosis<br>☐ Scoliosis<br>○ Left Convex ○ Right Convex |
| Eyes | ○ Not examined<br>○ Normal<br>○ Abnormal<br>o Ptosis<br>o Oculogyric crisis<br>o Episodes of gaze deviation<br>o Cortical blindness<br>o Nystagmus<br>o Strabismus<br>o Optic atrophy<br>o Retinal abnormality<br>Other abnormalities (please specify__________________)<br><br>Visual Acuity<br>○ Not examined<br>○ Normal<br>○ Abnormal<br><br>Wears glasses? ○ Yes ○ No<br>Currently on Vigabatrin? ○ Yes ○ No |

| | |
|---|---|
| Reflexes (scale, 0-4) | ◯ Not examined<br>◯ Normal<br>◯ Abnormal |

| | 0 | Trace | 1+ | 2+ | 3+ | 4+ |
|---|---|---|---|---|---|---|
| ☐ Right Biceps | ◯ | ◯ | ◯ | ◯ | ◯ | ◯ |
| ☐ Left Biceps | ◯ | ◯ | ◯ | ◯ | ◯ | ◯ |
| ☐ Right Triceps | ◯ | ◯ | ◯ | ◯ | ◯ | ◯ |
| ☐ Left Tricep | ◯ | ◯ | ◯ | ◯ | ◯ | ◯ |
| ☐ Right Brachioradialis | ◯ | ◯ | ◯ | ◯ | ◯ | ◯ |
| ☐ Left Brachioradialis | ◯ | ◯ | ◯ | ◯ | ◯ | ◯ |
| ☐ Right Patellar | ◯ | ◯ | ◯ | ◯ | ◯ | ◯ |
| ☐ Left Patellar | ◯ | ◯ | ◯ | ◯ | ◯ | ◯ |
| ☐ Right Gastrocnemius | ◯ | ◯ | ◯ | ◯ | ◯ | ◯ |
| ☐ Left Gastrocnemius | ◯ | ◯ | ◯ | ◯ | ◯ | ◯ |

Right Plantar: ◯ + ◯ - ☐

Left Plantar: ◯ + ◯- ☐

## GROSS MOTOR ABILITIES

| Gross Motor Abilities | |
|---|---|
| Gross Motor Abilities (Age up to 2nd Birthday) | ○ Not examined<br>○ Normal.<br>○ Abnormal<br><br>○ Level I (mild limitations): Sits on the floor, crawls, holds onto and "cruises" furniture, and holds objects in both hands without assistance.<br>○ Level II (mild to moderate limitations): Relies on hands or the assistance of others when sitting upright on the floor. Can crawl on hands and knees.<br>○ Level III (moderate limitations): Needs continuous lower back support and assistance in order to sit up, but able to move forward ("creep") while prone (on the abdomen).<br>○ Level IV (moderate to severe limitations): Able to roll back and forth without assistance, but needs adult help to sit up.<br>○ Level V (severe limitations): Voluntary control of limbs is limited. Requires assistance in sitting up, rolling over, and holding the head up. |
| Gross Motor Abilities (2 years - 4th Birthday) | ○ Not examined<br>○ Normal<br>○ Abnormal<br><br>○ Level I (mild limitations): Some delay or hypotonia (low muscle tone). Walks without assistance, and can sit easily while holding objects in both hands without assistance.<br>○ Level II (mild to moderate limitations): Requires assistance while sitting upright on the floor, may begin to hold himself/herself up via furniture and cruise (walks along furniture).<br>○ Level III (moderate limitations): Crawling is the preferred method of moving around, using hands and knees. Sits up unsupported, but in a "W" position with knees rotated and legs facing backwards.<br>○ Level IV (moderate to severe limitations): Sits up alone, but needs to use hands and arms to maintain position. Needs adaptive equipment for sitting and standing. Crawling preferred method for movement.<br>○ Level V (severe limitations): Extremely limited mobility. Needs |
| Gross Motor Abilities (4 years - 6th Birthday) | ○ Not examined<br>○ Normal<br>○ Abnormal<br><br>○ Level I (mild limitations): Some balance and coordination difficulty. Sits down and stands up without assistance; climbs stairs; emerging ability to run and jump.<br>○ Level II (mild to moderate limitations): Needs help with walking (aside from short distances) and may need to hold onto a table or furniture. Unable to skip, hop, or jump. Able to sit in a chair without assistance and climb stairs with assistance (railing).<br>○ Level III (moderate limitations): Walking requires assistance, either from an adult or a mobility assistance device. Sits with trunk support, |

| | |
|---|---|
| | lifts self up while holding onto something sturdy; can climb stairs with assistance.<br>o Level IV (moderate to severe limitations): Children sit on a chair but need adaptive seating for trunk control and to maximize hand function. Children move in and out of chair sitting with assistance from an adult or a stable surface to push or pull up on with their arms. Children may at best walk short distances with a walker and adult supervision but have difficulty turning and maintaining balance on uneven surfaces. Children are transported in the community. Children may achieve self-mobility using a powered wheelchair.<br>o Level V: Physical impairments restrict voluntary control of movement and the ability to maintain antigravity head and trunk postures. All areas of motor function are limited. Functional limitations in sitting and standing are not fully compensated for through the use of adaptive equipment and assistive technology. At Level V, children have no means of independent movement and are transported. |
| Gross Motor Abilities (>6 years) | o Not examined<br>o Normal<br>o Abnormal<br><br>o Level I (mild limitations): Speed, balance, and coordination are reduced. Runs, jumps, climbs stairs, and walks without assistance.<br>o Level II (mild to moderate limitations): Minimal ability to run and jump. Needs assistance when climbing steps (needs railing) or walking on uneven surfaces or an incline. Otherwise able to walk indoors and outdoors with little to no help.<br>o Level III (moderate limitations): Able to walk indoors and outdoors with the assistance of a mobility device (ex - walker, cane). Climbs stairs using handrails or with assistance. Cannot walk long distances.<br>o Level IV (moderate to severe limitations): Using mobility assistance, such as wheelchair, more so than before.<br>o Level V (severe limitations): Children are transported in a manual wheelchair in all settings. Children are limited in their ability to maintain antigravity head and trunk postures and control arm and leg movements. Transfers require complete physical assistance of an adult. At home, children may move short distances on the floor or may be carried by an adult. Children may achieve selfmobility using powered mobility with extensive adaptations for seating and control access. |

| Fine Motor |
| --- |
| ◯ Not examined<br><br>◯ Normal<br><br>◯ Abnormal<br><br>◯ Level I (mild limitations): Handles objects easily and successfully but slight limitation with precision and hand coordination. May need adult assistance when handling objects compared to other children of the same age. Limited in holding very small, very heavy, or fragile objects.<br><br>◯ Level II (mild to moderate limitations): Handles most objects, but with somewhat reduced quality or speed. May use alternative strategies, such as using only one hand instead of both or switching hands, or use a hard surface for support instead of both hands.<br><br>◯ Level III (moderate limitations): Handles objects with difficulty and slowly; often needs help by having objects placed in front of him/her.<br><br>◯ Level IV (moderate to severe limitations): Handles a limited selection of objects; needs constant adult help; at best, can perform simple actions: grasping or releasing objects.<br><br>◯ Level V (severe limitations): Does not handle objects and has severely limited ability to perform even simple actions. At best, can push, touch, press, or hold on to a few simple items. |

## BEHAVIOUR AND PSYCHIATRIC SYMPTOMS

| | |
|---|---|
| Psychiatric symptoms | ◯ Not examined<br>◯ Normal<br>◯ Abnormal (please fill in **"Behaviour questionnaire"**) |

| | Mild | Moderate | Severe |
|---|---|---|---|
| ☐ Aggression | ◯ | ◯ | ◯ |
| ☐ Anxiety | ◯ | ◯ | ◯ |
| ☐ Flat affect | ◯ | ◯ | ◯ |
| ☐ Hallucinations | ◯ | ◯ | ◯ |
| ☐ Impulsive | ◯ | ◯ | ◯ |
| ☐ Inattentive | ◯ | ◯ | ◯ |
| ☐ Labile | ◯ | ◯ | ◯ |
| ☐ Manic | ◯ | ◯ | ◯ |
| ☐ Paranoid/delusions | ◯ | ◯ | ◯ |
| ☐ Stereotypic movements | ◯ | ◯ | ◯ |

Natural History of SYNGAP1 encephalopathy

# MRI

Patient (ID): ..........................................    Date: ...... /....... /........ (DD/MM/YYYY)

Date of investigation: ...... /....... /........ (DD/MM/YYYY)

| **1. Cortex, CSF spaces, haemorrhage** *(Choose one of each)* | | | | |
|---|---|---|---|---|
| Cortex | o no abnormality | o mild-moderate atrophy or mild/moderate signal | o severe atrophy | o Abnormal sulcation of the cerebral cortex |
| | o Polymicrogyria | o Other malformation of cortical development, specify ______________ | o not known/ not done | o other ________________ |
| External CSF spaces | o no abnormality | o mildly-moderately widened | o strongly widened | o not known/ not done |
| Ventricles | o no abnormality | o mildly-moderately widened | o strongly widened | o not known/ not done |
| Subdural effusions | o none | o unilateral | o bilateral | o not known/ not done |

| **2. Deep gray matter structures** *(Choose one of each)* | | | | | |
|---|---|---|---|---|---|
| Putamen | o no abnormality | o T2 hyperintensity | o T2 hyperintensity plus atrophy | o not known/ not done | o dysplastic |
| Caudate | o no abnormality | o T2 hyperintensity | o T2 hyperintensity plus atrophy | o not known/ not done | o dysplastic |

| | | | | | |
|---|---|---|---|---|---|
| Pallidum | o no abnormality | o T2 hyperintensity | o T2 hyperintensity plus atrophy | o not known/ not done | o dysplastic |
| Thalamus | o no abnormality | o T2 hyperintensity | o T2 hyperintensity plus atrophy | o not known/ not done | o dysplastic |

| **3. White matter** *(Choose one of each)* | | | | |
|---|---|---|---|---|
| Myelination delay | o yes | if yes, please specify age of myelination ____ years ____ months If yes, amount of delay ____ years ____ months | o no | o not known/ not done |
| Abnormal T2 / FLAIR signal clearly different from unmyelinated white matter and not corresponding to the so-called terminal zones of myelination | o yes | if yes, please specify age of myelination ____ years ____ months If yes, amount of delay ____ years ____ months | o no | o not known/ not done |

| **4. Corpus callosum and hippocampi involvement** (Choose one of each) | | | | |
|---|---|---|---|---|
| o no | o thinned corpus callosum | o incomplete corpus callosum | o Abnormal hippocampi | o not known/ not done |

| **5. Infratentorial structures** (Choose one of each) | | | | |
|---|---|---|---|---|
| Cerebellum | o normal | o abnormal | o not known/ not done | |
| Hemisphere | o no abnormality | o T2 hyperintensity | o atrophy | o not known/ not done |
| Vermix | o no abnormality | o T2 hyperintensity | o atrophy | o not known/ not done |
| Brainstem involvement | o no abnormality | o abnormal T2/FLAIRhyperintensity | o atrophy | o not known/ not done |

Natural History of SYNGAP1 encephalopathy

# Laboratory Tests

Patient (ID): ………………………………………

Date: …… /……. /…….. (DD/MM/YYYY)

## Section 1: CSF

Date of investigation: …… /……. /…….. (DD/MM/YYYY)

| Was CSF collected? | o Yes | o No |
|---|---|---|

| Metabolic tests | Unit | Tested | Result (if tested) |
|---|---|---|---|
| Glucose level | mg/ml | o Yes o No | |
| CSF/serum glucose ratio | NV>0,6 | o Yes o No | |
| Total GABA (CSF) | nmol/mL | o Yes o No | |
| 5-Hydroxyindoleacetic acid (CSF) | nmol/L | o Yes o No | |
| Homovanillic acid (CSF) | nmol/L | o Yes o No | |
| HVA/5-HIAA ratio | | o Yes o No | |
| L-Serine (CSF) | nmol/mL | o Yes o No | |
| Other | Enter unit: | o Yes o No | |
| Other | Enter unit: | o Yes o No | |
| Other | Enter unit: | o Yes o No | |

## Section 2: Skin Biopsy

| Was a skin biopsy collected? | o Yes | o No |
|---|---|---|

## Section 3: Biobank

| Was a sample in Biobank stored? | | |
|---|---|---|
| Plasma | o Yes | o No |
| Serum | o Yes | o No |
| CSF | | |

Natural History of SYNGAP1 encephalopathy

# EEG

Patient (ID): ……………………………………..

Date of investigation: …… /……. /…….. (DD/MM/YYYY)

Age of Subject: _____ years_____ months

1. Awake State

   - Posterior dominant rhythm _____ Hz
   - Normal Features
   - Background Slowing
     - Focal
     - Regional
     - Hemispheric
     - Diffuse

2. Sleep State
   - Sleep state not recorded
   - Normal Features
   - Abnormal Features

   If Abnormal, describe: ______________________________________________

3. Epileptiform Abnormalities
   - Focal Spikes Location: ______________________________
   - Bilateral Independent Spikes Location: ______________________________
   - Multifocal Spikes Location: ______________________________
   - Generalized Spike and Wave Location: ______________________________
   - Sharps Location: ______________________________
   - Periodic Complexes Location: ______________________________
   - CSWS Location: ______________________________
   - Photoparoxysmal response Location: ______________________________
   - Ictal Event(s) Location: ______________________________
   - Other Describe:_______________________________

4. Quantification of Epileptiform Abnormalities
    - Continuous (>90% of record)
    - Abundant (50-89% of record)
    - Frequent (10-49% of record)
    - Occasional (1-9% of record)
    - Rare (< 1 % of record)

Natural History of SYNGAP1 encephalopathy

# Treatment (medications and non-pharmaceuticals)

Patient (ID): ……………………………………..      Date: …… /……. /…….. (DD/MM/YYYY)

Current Body Weight: _________kg*

**CURRENT TREATMENT**

| Current Treatment | Dose (mg/d) | Comment (free text) |
|---|---|---|
| Ketogenic diet | | |
| ◯ Yes ◯ No | If yes: Ratio of fat to non-fat calories _____: _____ | |
| Selective serotonin reuptake inhibitor (SSRI) drugs | | |
| o Yes (please specify)<br>o No | | |
| Tricyclic drugs | | |
| o Yes (please specify)<br>o No | | |
| Stimulant drugs | | |
| o Yes (please specify)<br>o No | | |
| Antipsychotic drugs | | |
| o Yes (please specify)<br>o No | | |
| Anticonvulsive drugs and other anticonvulsive treatment | | |
| o Yes (please specify)<br>o No | | |
| Valproate | | |
| Phenytoin | | |
| Felbamate | | |
| Perampanel | | |
| Levetiracetam | | |
| Lamotrigine | | |

| | | |
|---|---|---|
| Clobazam | | |
| Sulthiame | | |
| Phenobarbital | | |
| Vigabatrin | | |
| Carbamazepine | | |
| Oxcarbazepine | | |
| Topiramate | | |
| Lacosamide | | |
| Zonisamide | | |
| Other Anticonvulsive | | |
| L-Serine | | |
| ○ Yes<br><br>○ No | | |
| Other drugs or supplements | | |
| ○ Yes (please specify)<br><br>○ No | | |
| Levothyroxine | | |
| Antibiotics | | |
| NSAIDs | | |
| Vitamins | | |
| Dietary Supplements | | |
| Melatonin | | |
| Clonidine | | |
| Coenzyme Q10 | | |
| Other drug 1: | | |
| Other drug 2: | | |
| Other Treatment | | |
| ○ Yes (please specify)<br><br>○ No | | |
| Speech Therapy | | |
| Occupational Therapy | | |
| Physical Therapy | | |
| Other 1: | | |

Non-compliance (diet and/or drug treatment) suspected

○ No

○ Yes (please specify: ______________________________________________________

**PREVIOUS TREATMENT**

| Previous Treatment | Dose (mg/d) | Period of treatment | Cause of withdrawal | Comment (free text) |
|---|---|---|---|---|
| Ketogenic diet | | | | |
| ◯ Yes ◯ No | If yes: Ratio of fat to non-fat calories ____: ____ | | | |
| Selective serotonin reuptake inhibitor (SSRI) drugs | | | | |
| o Yes (please specify)<br><br>o No | | | | |
| Tricyclic drugs | | | | |
| o Yes (please specify)<br><br>o No | | | | |
| Stimulant drugs | | | | |
| o Yes (please specify)<br><br>o No | | | | |
| Antipsychotic drugs | | | | |
| o Yes (please specify)<br><br>o No | | | | |
| Anticonvulsive drugs and other anticonvulsive treatment | | | | |
| o Yes (please specify)<br><br>o No | | | | |
| Valproate | | | | |
| Phenytoin | | | | |
| Felbamate | | | | |
| Perampanel | | | | |
| Levetiracetam | | | | |
| Lamotrigine | | | | |
| Clobazam | | | | |
| Sulthiame | | | | |
| Phenobarbital | | | | |
| Vigabatrin | | | | |
| Carbamazepine | | | | |
| Oxcarbazepine | | | | |
| Topiramate | | | | |
| Lacosamide | | | | |

| | | | | |
|---|---|---|---|---|
| Zonisamide | | | | |
| Other Anticonvulsive | | | | |
| L-Serine | | | | |
| ○ Yes<br><br>○ No | | | | |
| Other drugs or supplements | | | | |
| ○ Yes (please specify)<br><br>○ No | | | | |
| Levothyroxine | | | | |
| Antibiotics | | | | |
| NSAIDs | | | | |
| Vitamins | | | | |
| Dietary Supplements | | | | |
| Melatonin | | | | |
| Clonidine | | | | |
| Coenzyme Q10 | | | | |
| Other drug 1: | | | | |
| Other drug 2: | | | | |
| Other Treatment | | | | |
| ○ Yes (please specify)<br><br>○ No | | | | |
| Speech Therapy | | | | |
| Occupational Therapy | | | | |
| Physical Therapy | | | | |
| Other 1: | | | | |
